# Benchmark Data Set of Crystalline Organic Semiconductors

**DOI:** 10.1021/acs.jctc.3c00861

**Published:** 2023-11-16

**Authors:** Andriy Zhugayevych, Wenbo Sun, Tammo van der Heide, Carlos R. Lien-Medrano, Thomas Frauenheim, Sergei Tretiak

**Affiliations:** † Max Planck Institute for Polymer Research, Ackermannweg 10, 55128 Mainz, Germany; ‡ Bremen Center for Computational Materials Science, Am Fallturm 1, 28359 Bremen, Germany; § Los Alamos National Laboratory, Los Alamos, New Mexico 87545, United States

## Abstract

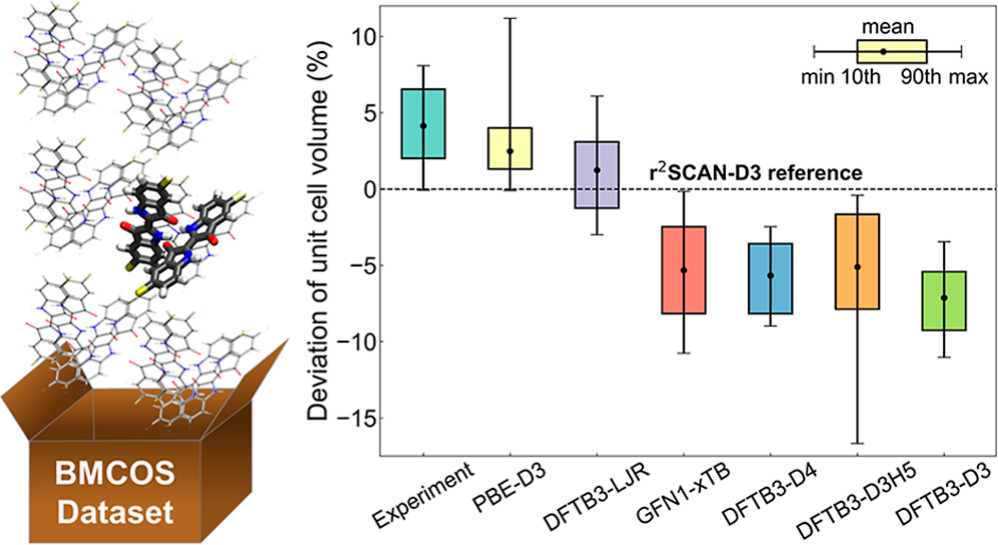

This work reports a Benchmark Data set of Crystalline Organic Semiconductors to test calculations of the structural and electronic properties of these materials in the solid state. The data set contains 67 crystals consisting of mostly rigid molecules with a single dominant conformer, covering the majority of known structural types. The experimental crystal structure is available for the entire data set, whereas zero-temperature unit cell volume can be reliably estimated for a subset of 28 crystals. Using this subset, we benchmark r^2^SCAN-D3 and PBE-D3 density functionals. Then, for the entire data set, we benchmark approximate density functional theory (DFT) methods, including GFN1-xTB and DFTB3(3ob-3-1), with various dispersion corrections against r^2^SCAN-D3. Our results show that r^2^SCAN-D3 geometries are accurate within a few percent, which is comparable to the statistical uncertainty of experimental data at a fixed temperature, but the unit cell volume is systematically underestimated by 2% on average. The several times faster PBE-D3 provides an unbiased estimate of the volume for all systems except for molecules with highly polar bonds, for which the volume is substantially overestimated in correlation with the underestimation of atomic charges. Considered approximate DFT methods are orders of magnitude faster and provide qualitatively correct but overcompressed crystal structures unless the dispersion corrections are fitted by unit cell volume.

## 1 Introduction

The unique optoelectronic properties of organic semiconductors arise from their delocalized π-conjugated electronic system. The task of rational design for these materials for particular applications is a formidable one due to the intricate electronic processes involved and complex structural motifs. In principle, the multiscale structural diversity of organic semiconductors^1^ offers great potential for fine-tuning their electronic properties. Nevertheless, achieving accurate and reliable results at a reasonable computational cost necessitates tailored modeling approaches that account for the complexity of their electronic structure and conformational motifs.^2,3^ Even for a crystalline morphology, there are uncertainties with atomic positions and polymorph energy ranking because of the omnipresence of various types of disorder and external factors influencing intermolecular packing.^4,5^ As a result, significant uncertainties emerge in computed properties, such as charge carrier mobility, as errors accumulate from multiple sources, including molecular geometry and force constant inaccuracies, electronic and vibronic couplings, and the accuracy of the electron–phonon Hamiltonian and its solution methods. Hence, the availability of a benchmark data set for organic semiconductors in their solid-state form is important for assessing the predictive capabilities of computational methods concerning the structural and electronic properties of these materials. This work is aimed to provide such a data set for single crystals, which not only incorporate the most appropriate structural morphology for this purpose but are also of practical use in optoelectronics.^5−8^ It should be noted that although a crystal structure can usually be determined experimentally, often materials modeling can be performed only for simulated structures. This includes, for example, crystalline π-conjugated polymers^9^ whose atomistic structure can be determined only by a combination of computational and various experimental approaches, including, e.g., vibrational spectroscopy.^10−12^

Over time, numerous large^13−16^ and small^17^ data sets of crystalline organic semiconductors have been accumulated. However, these data sets were not specifically designed for benchmarking purposes. On the other hand, available benchmark data sets for molecular crystals^18−22^ and clusters^23,24^ do not contain sufficient samples of extended π-conjugated molecules commonly used in optoelectronic applications. Alternatively, crystals for benchmarking can be taken directly from the Cambridge Structural Database (CSD) or similar heterogeneous sources.^25^ However, practitioners should be aware of possible ambiguity in the preprocessing of the raw structural information before benchmarking.

This work introduces the BMCOS1 data set (Benchmark Data set of Crystalline Organic Semiconductors part 1, https://cmsos.github.io/bmcos/) containing 67 crystals, whose molecular structures are explicitly listed in Figures S1 and S2 and Table S8. The index 1 reflects the selection rules for crystals included in the data set: the BMCOS1 is aimed to cover all major classes of single-conformer molecules studied experimentally for potential use in optoelectronics, which have a well-characterized dominating disorder-free crystalline polymorph. Here, a “single-conformer” means that only a single dominant molecular conformation is observed at ambient conditions in solution or the solid state, thus eliminating intramolecular degrees of freedom in the identification of crystal packing. In this sense, flexible dihedrals are acceptable only if the equilibrium geometry is near-planar and rotations have a high energy penalty, e.g., oligothiophenes. Furthermore, for the convenience of benchmarking, the chemical space is limited to the first three rows of the Periodic Table, and the size of the unit cell is capped by about 100 atoms. Moreover, to make the data set suitable for any further analysis of electronic properties, e.g., mapping the wave function of the crystal to molecular orbitals of a symmetric molecule in vacuum, all crystal structures are kept in a unified form: atoms are unfolded to the unit cell, molecules are connected and have the same numbering across the data set, and symmetry-inequivalent molecules are classified by space group orbits.

The selection of chemical moieties to be included in the data set reflects motifs observed in organic semiconductors. Pure hydrocarbons in BMCOS1 are represented mainly by polycyclic aromatic hydrocarbons, including acenes. Sulfur atoms in organic semiconductors occur mainly in the thiophene ring; therefore, BMCOS1 has enough coverage of thienoacenes and oligothiophenes. Tetrathiafulvalene is another sulfur-based π-conjugated moiety included in the data set. Nitrogen is represented by azaacenes, imides, and cyanocarbons. Oxygen is observed mainly in quinone-like structures, though the furan ring and bridging oxygen are also included. Halogens are commonly used in organic semiconductors to tune their properties by hydrogen substitutions, and such molecules are added to BMCOS1 as well. Other nonheavy elements are rare in organic electronics; therefore, we include one molecule per such element: phosphorus in triphenylphosphine, silicon in spirobidibenzosilole, and boron in BODIPY. Branching molecules are usually large in size so we include only their core moieties, such as triphenylene, triphenylbenzene, triptycene, triphenylphosphine, and spirobifluorene, despite these crystals having more than 100 atoms in the unit cell. The largest system included in BMCOS1 is fullerene C60: its 240 atoms in the unit cell substantially exceed the median size of the data set, but it is one of the most commonly used organic semiconductors and represents an important structural motif. On the other side, several small molecules that form semiconductors only as part of larger molecules are added to BMCOS1, in part to make a connection to existing small-molecule data sets, high-level calculations, and extensive experimental studies. The resulting size of the BMCOS1 is consistent with similar data sets for efficient benchmarking by a wide range of methods.

We use density functional theory (DFT) at the r^2^SCAN-D3 level as the main reference method for BMCOS1. This model incorporates the popular DFT-D3 empirical dispersion correction.^26^ The more advanced r^2^SCAN-D4 should potentially be even more accurate for molecular crystals.^27^ However, not all software packages have D4 dispersion corrections implemented, whereas, for the majority of organic molecules, D3 and D4 models demonstrate comparable performance.^28^ The commonly used PBE-D3 density functional is much faster than r^2^SCAN-D3; therefore, it is considered a second reference method. PBE-D3 is particularly important for crystals with hundreds of atoms in the unit cell^29^ and for the calculation of force constants, as it might be the top choice DFT-D framework combining reasonable accuracy with practical utility. Hybrid functionals such as CAM-B3LYP-D3 usually provide superior accuracy for both intra- and intermolecular geometries of π-conjugated systems.^9,30^ However, these techniques are computationally prohibitive under periodic boundary conditions. Finally because the typical accuracy of both PBE-D3 and r^2^SCAN-D3 is often higher than the accuracy of extrapolation of available experimental data to zero temperature, the latter cannot serve as a reliable reference geometry for the majority of systems from BMCOS1.

In practice, a single-crystal, disorder-free morphology is rarely observed in organic semiconducting materials, so large supercells are typically needed to obtain a realistic model of a material. Approaching those scales requires computationally efficient and scalable schemes. In this work, we therefore benchmark two classes of approximate DFT methods augmented with dispersion corrections: the Density Functional Tight Binding (DFTB)^31,32^ and Geometry, Frequency, Noncovalent, eXtended Tight Binding (GFN-xTB)^33,34^ approaches. Among the various DFTB approaches available, we select the DFTB3 method^35^ with the PBE-based 3ob-3-1 parametrization^36^ covering most elements contained in BMCOS1. Multiple studies have shown that the DFTB3 with dispersion corrections provides a relatively accurate description of organic molecular crystals.^37−39^ For the xTB approach, we choose the GFN1-xTB Hamiltonian,^34^ providing a relatively accurate description of molecular systems.^33^ The GFN2-xTB^40^ has been under investigation as well, but we find that many systems from BMCOS1 either relax to unphysical geometries or experience convergence issues arising from the aforementioned fact, preventing us from discussing this method in detail.

The text is organized as follows: we start with a description of the computational methodology used and discuss molecular systems constituting the BMCOS1 data set. We then compare DFT-D results for a subset of BMCOS1 with extensive experimental data and benchmark approximate DFT methods against DFT-D for the entire BMCOS1 data set. Finally, we summarize our observations and conclude.

## 2 Methodology

### 2.1 Reference Computational Methods

All DFT-D calculations of crystals are performed with projector augmented wave (PAW) pseudopotentials as implemented in the VASP program^41^ with a 900 eV energy cutoff. For a smaller cutoff, “pulay stress” errors in volume and elastic tensor may become significant. Nevertheless, exploring a reasonable decrease in the cutoff might be helpful for large systems. Therefore, we have performed calculations with a 600 eV cutoff, where the equilibrium unit cell volume is obtained by fitting the Murnaghan equation of state, whereas all other degrees of freedom are relaxed at a fixed volume. We use a fixed (per crystal) Γ-centered *k*-grid with 0.2 Å^–1^ spacing and 0.2 eV Gaussian smearing. The final energy is calculated with a finer *k*-grid with 0.1 Å^–1^ spacing and the tetrahedron method with Bloch corrections. In all calculations, we use PREC = accurate setting. For the purpose of benchmarking, we have overtightened the convergence criterion for forces to 1 meV/Å, which necessarily requires hundreds of geometry relaxation steps for molecules from the BMCOS1 set. To reach the desired accuracy in forces, the electronic energy tolerance has been set to 0.1–1 μeV (the lower value guarantees accurate forces in cross-checking single-point r^2^SCAN-D3 calculations). Furthermore, the Gaussian smearing parameter has been reduced to 0.05 eV for narrow-gap systems such as hexacene. Normally, VASP-default accuracy for electronic energy of 0.1 meV and a slightly decreased threshold for change in total energy during relaxation to 0.1 meV give optimal geometry as accurate as the method itself within less than 100 iterations. The final forces on atoms then typically converge to an order of tens of meV/Å. The primitive cell is used with a predefined basis of primitive vectors unless such an autogenerated shape is too elongated (e.g., chrysene crystal), in which case the lattice reduction is performed. Bulk and shear moduli are derived from the elasticity tensor, according to ref (42).

DFT calculations of molecules in a vacuum are performed using the Gaussian 16 program.^43^ As the reference method for single-molecule properties, we use the CAM-B3LYP/Def2-TZVP model chemistry that previously demonstrated robust performance for organic semiconductors.^30^

### 2.2 Approximate DFT Methods

DFTB and xTB calculations for molecules and crystals are performed using the DFTB+ software package.^44^ The first Brillouin zone is sampled by a Γ-centered *k*-grid of at least 0.2 Å^–1^ spacing. Self-consistent charge cycles are performed until the total energy converges to 0.1 μHa (3 μeV). Geometry relaxation is stopped when the maximum absolute gradient component becomes smaller than 0.1 mHa/Bohr (5 meV/Å). We explore four types of dispersion corrections added to DFTB3. Namely, the D3,^45^ D4,^28^ and DFTB3-D3H5^46^ methods as implemented in the DFTB+ package. Additionally, we explore the Lennard-Jones (LJ) potential^47^ parametrized as follows: the initial set of LJ parameters (element-wise distances and energies) is taken from the Universal Force Field^48^ (UFF), listed in Table S1. Then, the LJ distances and energies are rescaled by 0.9608 and 0.7970 to match the r^2^SCAN-D3 equilibrium unit cell volume and binding energy, respectively, for the BMCOS1 data set (Table S2 and Figures S11 and S12). Consequently, we distinguish four DFTB methods: DFTB3-D3, DFTB3-D4, DFTB3-D3H5, and DFTB3-LJR (rescaled UFF). Their parametrization is detailed in Section S3. Following ref,^35^ all third-order DFTB3 calculations, except for DFTB3-D3H5, employ a damped γ-function (exponent 4.0) of the Coulomb repulsion between density fluctuations for all interactions involving hydrogen. In the case of DFTB3-D3H5, the hydrogen–hydrogen repulsion per ref (46) has been activated. For GFN1-xTB, we use the standard parametrization described in ref (34).

### 2.3 Geometry Processing

Initial geometries are taken from the CSD database, except for dibenzoindigo. In the case of multiple CSD entries, we select geometry measured at a lower temperature and by a higher-level method. Original crystallographic information files are processed by the same algorithm and reduced to a canonical form (as described in the BMCOS project webpage). In particular, molecules have the same numbering across the data set to reduce their superposition to simple alignment^49^ instead of atom matching.^50^ Molecules and crystal geometries are saved in the XYZ file format. In the latter case, the entire Bravais unit cell is stored, translation vectors are denoted by “Tv,” and the complete symmetry information is given in the comment line.

### 2.4 Benchmarking Details

For method comparison, we use multiple parameters: the complete list of definitions is given in Section S1, whereas parameters explicitly discussed in the text are also defined here. The first quantity to compare is the unit cell volume per atom ‘V1′; its relative deviation “dV1” is defined as *V*1/*V*1_ref_ – 1, where “ref” labels a reference method. Deviation in the unit cell shape “dSh” is defined as , where *T* is the matrix of translation vectors, the norm is Frobenius, and the matrices are superimposed to minimize the norm. Translations and rotations of individual molecules relative to a reference are denoted as displacement “dr” and deflection angle “phi.” The difference in the intramolecular geometry is denoted as “dev” and is calculated as the root-mean-square deviation of atom positions excluding hydrogens. Here, both molecules are superimposed to minimize this deviation. Finally, the binding energy per molecule “Eb” is calculated with respect to a fully relaxed molecule in vacuum (planar conformation is taken for nonrigid molecules). Relative and dimensionless deviations are given in percents. For all scalar and vector quantities, we calculate the “method error” over the data set using several scalar parameters (“vectors are treated as sequences of independent scalars): mean value (“mean,” “ave”), root-mean square value, 90th percentile (“90th”), and maximum value (“max”). The BMCOS project webpage (https://cmsos.github.io/bmcos/) contains the BMCOS1 data set files and other relevant information.

## 3 Structural Types in BMCOS1 Data Set

The BMCOS1 data set is aimed to cover all structural types of potentially high-performing organic semiconductors consisting of relatively small molecules (tens of atoms) without nonconjugated side chains. First and foremost, the herringbone packing of acenes and heteroacenes, such as pentacene and DNTT (dinaphthothienothiophene), enables superior two-dimensional charge carrier mobility.^7^ There are several subtypes of this motif^25^ covered in the data set, including one interdigitated structure IF12b (6,12-dihydroindeno[1,2-*b*]fluorene), which potentially might increase the dimensionality of the electronic connectivity network.

The columnar packing of slipped π-stacks is another commonly observed crystalline motif for conjugated molecules.^51^ This structure fosters a large and robust intermolecular electronic coupling network along the stacks, whereas the interstack couplings are typically small. This class in the BMCOS1 data set is represented by several systems with different levels of interdigitation between stacks, ranging from coronene to NDI (naphthalenetetracarboxdiimide).

Hydrogen-deficient (H-poor) molecules bring forward another type of intermolecular contacts in terms of electronic couplings. Here, the limiting cases are fullerenes and TCNQ-F4 (tetrafluoro-tetracyanoquinodimethane), which have no hydrogen atoms. Instead, the surface of such molecules is terminated with π-conjugated atoms and functional groups^52^ such as halogens, oxygen as in quinones, cyano (CN), and nitro (NO_2_) groups. All of these terminations are represented in BMCOS1. Further, aromatic nitrogen and bridging chalcogens (e.g., in thiazolothiazole) stand at the molecular surface, forming unobscured intermolecular contacts between π-conjugated segments. Consequently, regardless of packing, H-poor molecules usually form small but numerous electronic contacts, which are non-negligible. This is especially the case for heavy electron-rich atoms like chalcogens and halogens, allowing for shortened interatomic contacts.^53,54^

Other known structural types are represented by larger molecules involving flexible conjugated backbones and solubilizing side chains, which are beyond the immediate scope of the BMCOS1 data set. This includes two important crystalline motifs: brickwork packing observed for TIPS-pentacene and wire mesh packing appearing for some acceptor–donor–acceptor molecules.^29^ Electronic connectivity in these crystals is conducted via a π-stack type of intermolecular contact, so that, partially, these materials are represented in BMCOS1. Further, several short flexible linear oligomers have been added, including oligo(thiophene) and oligo(*p*-phenylenevinylene) motifs. Flexible branching oligomers have conformational complexity, which typically leads to high disorder and low charge carrier mobility. Nevertheless, they are widely used as charge transporting layers (e.g., as host materials) in organic electronics.^55^ In principle, there are no fundamental limitations to their performance as semiconductors. Therefore, we have included in our data set some small representatives of this class, such as triphenylbenzene, and only branching core moieties, such as benzotrithiophene.

Across a broad variety of structural motifs observed for molecular crystals, some materials have large electronic couplings but were never recognized as semiconductors,^56^ thus motivating exploration of these structural types of organic semiconductors. As such, we have included two more classes of molecules sparsely studied for organic electronics. The first one has rigid (fused) branching or star-shaped molecules, represented by tetrathienophenazine.^57^ The other includes nonplanar (nonfullerene) π-conjugated molecules, triptycene, and spirobifluorene.

## 4 Benchmarking of Reference DFT-D Methods Against Experimental Data

**Figure 1:**
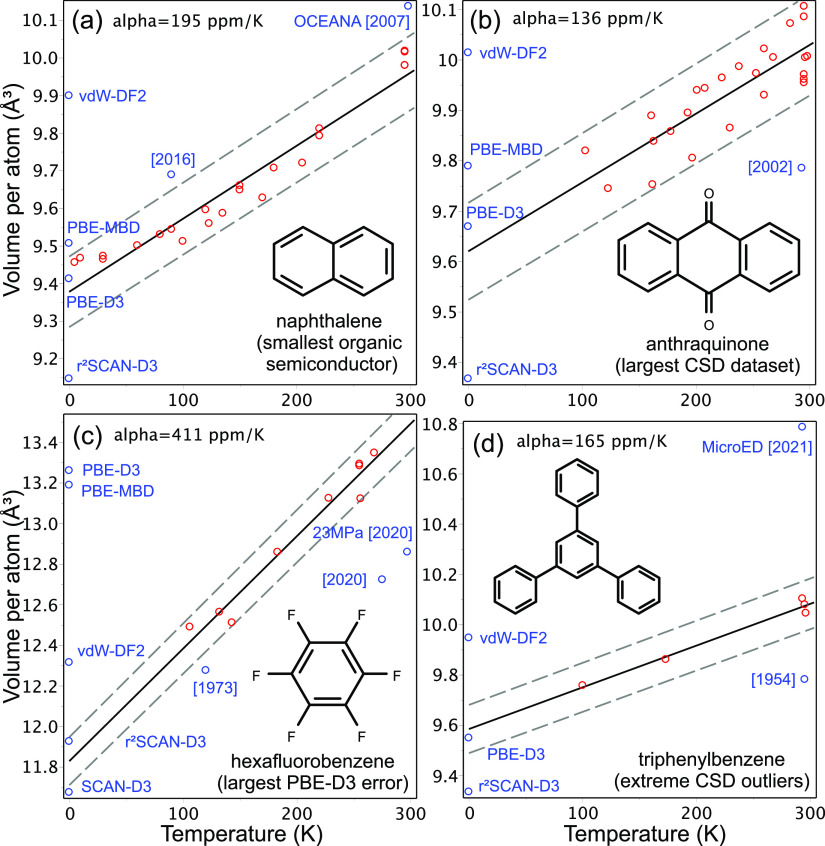
Thermal expansion of the selected molecular crystals. Dashed lines mark a 1% deviation. Red dots show experimental data from the CSD database used for linear fit. Blue dots are outliers (publication year is given) and DFT calculations (at 0 K).

DFT-D benchmarking for relaxed optimized geometries requires a reference low-temperature experimental crystal structure corrected for quantum effects, which might be around 1% in volume^58^ and even larger for large-amplitude modes.^30^ Since it is rarely available for molecular semiconductor crystals, we only test the unit cell volume extrapolated to 0 K from elevated temperatures. Such extrapolation can be reliably performed for 28 crystals from the BMCOS1 data set (these have at least 3 data points in a broad temperature range within 1% deviation from a line, Table S9). For C60, both rotationally ordered and disordered polymorphs are used in the extrapolation since their thermal coefficients are statistically indistinguishable for the available data. Because the temperature range is relatively small, resulting in only a ∼5% volume change at 300 K, we use a linear fit. Thermal expansion data for selected crystals are summarized in Figure 1. Deviations of available experimental data at a fixed temperature are usually smaller than 1%, whereas outliers come from older data and less accurate measurement techniques. Derived thermal expansion coefficients agree well with studies on large data sets.^59^ Subsequently, a value of 150 ppm/K can be taken as a rough universal coefficient for the entire BMCOS1 data set. Modeling of thermal expansion and thermodynamic quantities is possible for simple molecular crystals,^60^ but it becomes computationally demanding for large unit cells.

**Figure 2:**
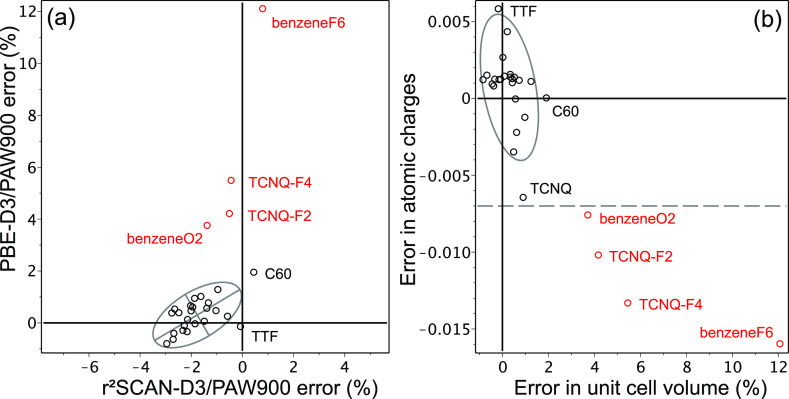
(a) r^2^SCAN-D3 and PBE-D3 errors for unit cell volume; see Table 1 for explanations. The ellipse is drawn for 2σ with red dots excluded. (b) Correlation plot for PBE-D3 errors for the unit cell volume vs its errors for the Hirshfeld atomic charges relative to CAM-B3LYP functional with TZVP basis.

Results of benchmarking of r^2^SCAN-D3 and PBE-D3 for the 28 crystals subset are shown in Figure 2 and Table 1. The r^2^SCAN-D3 systematically underestimates the unit cell volume by 1–2% in consistency with the ref (27) conclusion that the r^2^SCAN-D4 slightly overbinds molecular crystals. Overall, it shows robust performance with a very small range of errors across the entire set of 28 systems. In contrast, PBE-D3 fails to predict the unit cells for two semiconductors, TCNQ-F4 and TCNQ-F2, substantially overestimating their volumes. To check if this error is related to the terminal electronegative atoms, we have added hexafluorobenzene (benzeneF6) and benzoquinone (benzeneO2) to the BMCOS1 data set. Markedly, PBE-D3 fails for these two molecular crystals as well. To be able to predict such failures, we notice that errors in the unit cell volume correlate well with errors in the Hirshfeld atomic charges, Figure 2b. As such, if we exclude molecules with large errors in the atomic charges (dashed line in Figure 2b), the performance of PBE-D3 on the reduced subset becomes superior except for C60; typically, it is accurate within 1% in the volume. Additional study on special data sets (e.g., ref (61)) is needed to pinpoint the problem. Evidently, the influence of intermolecular halogen contacts is critical: the PBE-D3 error for trichlorotrifluorobenzene is similar to that for hexafluorobenzene, whereas for trifluorobenzene, it is substantially smaller, and the error in volume for triazine is vanishing. Interestingly, for narrow-gap molecules such as long acenes,^62^ PBE-D3 produces accurate intermolecular geometry despite predicting very inaccurate electronic structure (see, e.g., calculated gaps in the BMCOS project webpage).

**Table 1:** Benchmarking DFT-D Against Experiment for the Unit Cell Volume for 28 Crystals From BMCOS1^a^

	r^2^SCAN-D3	PBE-D3	subset
median	–1.8	0.4	0.4
mean	–1.6	1.2	0.3
standard dev.	1.0	2.6	0.6
lower decile	–2.7	–0.4	–0.5
upper decile	–0.1	4.1	1.1
min	–2.9	–0.8	–0.8
max	0.8	12.1	1.9

a Available experimental data in the CSD data set are extrapolated to 0 K. All numbers are deviations in percent. The “subset” column shows PBE-D3 data with four crystals excluded based on PBE errors in atomic charges (red dots in Figure 2b). See details in Table S10

**Figure 3:**
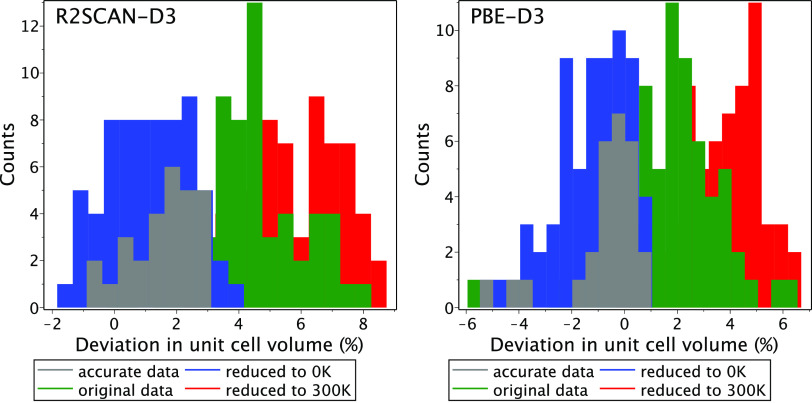
Relative difference between the unit cell volume derived from experimental data and its value relaxed with the r^2^SCAN-D3 and PBE-D3 methods. Different sets correspond to different treatments of thermal expansion. In “accurate data,” we use the extrapolated to 0 K volume available for a subset of 28 crystals. In “original data,” thermal expansion is ignored, whereas in “reduced” data, the geometries are extrapolated to a given temperature by the best-estimated thermal expansion coefficients. The latter are either derived from the available experimental data (37 crystals, including the “accurate data” subset) or set to 150 ppm/K. Out of scale are benzene at 300 K and benzeneF6 at 0 K for PBE-D3, and benzene, benzeneF6, benzeneO2, and BODIPY at 300 K for r^2^SCAN-D3.

A comparison of calculated and measured crystal structures for the entire BMCOS1 data set can only be made given the uncertainty of thermal expansion effects, which are usually anisotropic and larger than the accuracy of DFT-D. In such comparisons, we will always set a specific DFT-D method as the reference because experimental data are obtained with different approaches and at different temperatures. For the unit cell volume, we can apply the aforementioned universal thermal expansion coefficient (150 ppm/K) to all crystals with insufficient data. The result is shown in Figure 3 and is consistent with the above study of 28 systems. Furthermore, it shows that overall thermal effects across the entire BMCOS1 data set are about a few percent in volume, that is about 1% on a linear scale. Neglecting these effects leads to a systematic bias clearly visible in the green and red histograms in Figure 3. Among other geometrical parameters, we can compare those that are less sensitive to thermal expansion to avoid any extrapolation to 0 K: the shape of the unit cell, the orientation of molecules, and intramolecular geometry (excluding hydrogens), using one scalar descriptor per each property (“dSh”, “phi,” and “dev,” respectively, as defined in Section S1). For both r^2^SCAN-D3 and PBE-D3, on average, the shape deviates by 1%, the orientation deflects by 1°, and the intramolecular geometry differs by tens of mÅ (Tables S11–S12). It should be noted that the large difference in intramolecular geometry for rigid molecules might indicate reduced fidelity of experimental data (e.g., the two outliers, dibenzoindigo and naphthodithiophene (NDT) in Figures S3–S4).

Modeling of molecular crystals has its own specific challenges^60^ which can be analyzed using the BMCOS1 data set. First of all, these are soft systems in the sense that many collective structural motions have a complex flat potential energy surface (PES). Moreover, there is no clear separation between these motions and the other nuclear degrees of freedom. Instead, there is a continuous distribution of vibrational frequencies, raising up to typical bond stretching values (without counting phonon dispersion). For our data set, the lowest frequencies at the Γ-point range from 1.5 meV for triptycene to 8 meV for TCNQ-F2 with one molecule per primitive cell (both molecules are rigid and relatively small). Three crystals have imaginary frequencies in PBE-D3 due to the brute-force use of finite differences. Linear PES scans for these modes show that they correspond to complex collective motions with energy changes as small as tenths of meV per unit cell upon mass-weighted displacement of 0.5 Å (Figures S5–S7). Similarly, the lowest eigenvalue of the elastic matrix^63^ is often very small. For PBE-D3, there is one crystal with a negative elastic modulus due to the use of finite differences: a relaxed PES scan along two unit cell parameters shows a positive-definite quadratic PES, but the energy changes only by tens of μeV per atom upon 1% deformation (Figure S8 and Table S4).

The BMCOS1 data set represents organic semiconductors with relatively small unit cells, whereas the majority of materials used in organic electronics have more complex structures. Therefore, it is important to have methods that are scalable to hundreds of atoms in the unit cell. This requirement immediately screens out the r^2^SCAN functional, which is slightly less accurate (though more reliable) and substantially slower than the PBE model (Figure S9). Next, the convergence criteria can be relaxed to a default tolerance for the wave function and 1–2 orders of magnitude tighter convergence for geometry relaxation, yielding geometries with subpercent accuracy (Table S15) and practically converged binding energies. In terms of the wave function, important parameters influencing computational cost are the plane-wave energy cutoff and k-grid spacing. The former is critical for unit cell optimization and elastic matrix calculation. In particular, unconstrained relaxation of the unit cell with a 600 eV cutoff systematically underestimates its volume by about 3%, which is substantially larger than the error of PBE-D3 (Table S16). Coarse k-grids produce meaningful results for organic semiconductors due to their large electronic band gaps and small band dispersions, especially for geometry relaxation: a grid with 0.2 Å^–1^ spacing gives final gradients essentially the same as those produced by finer grids (Table S17). Smaller k-grids are still acceptable within the accuracy of DFT-D itself up to 2 × 2 × 2 grid, corresponding to the spacing of about 0.5 Å^–1^ for small unit cells (Table S18). At the same time, ignoring electronic dispersion in directions where molecules have close contact with their periodic images leads to poor results (Tables S7 and S18). This is valid for most systems except probably for long molecules such as pentacene, where the precise position of the herringbone layers with respect to each other is no longer important.

## 5 Benchmarking of Approximate DFT Methods Against DFT-D

For benchmarking the approximate DFT methods, we take the r^2^SCAN-D3 results as the reference because this method is more reliable than the PBE-D3 model. Notably, the typical difference between these two DFT-D approaches for calculated geometries (Table S13) is much smaller than the difference between the reference and the approximate DFT methods. In this section, the complete data set has been employed for benchmarking. However, DFTB methods exclude BODIPY and spirobidibenzosilole due to the lack of boron and silicon parameters in the 3ob-3-1 set.

**Figure 4:**
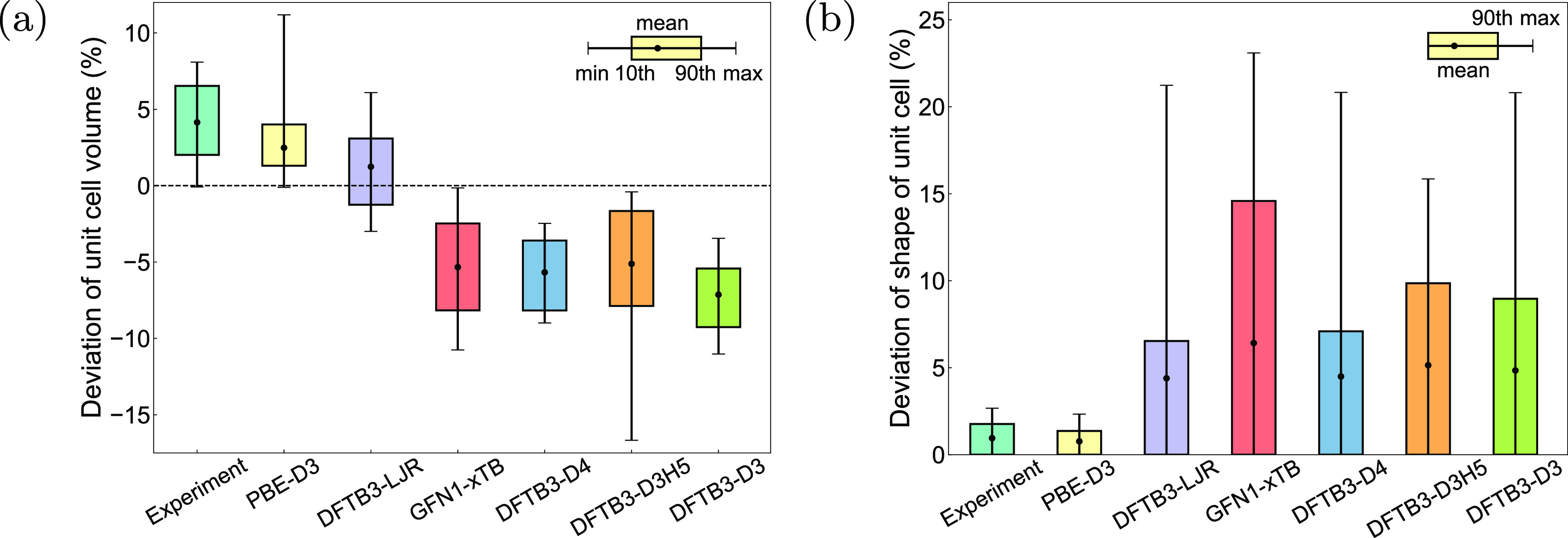
Benchmark of approximate DFT methods against r^2^SCAN-D3 for (a) unit cell volume and (b) shape. Here, the experimental data are not extrapolated to zero temperature.

**Figure 5:**
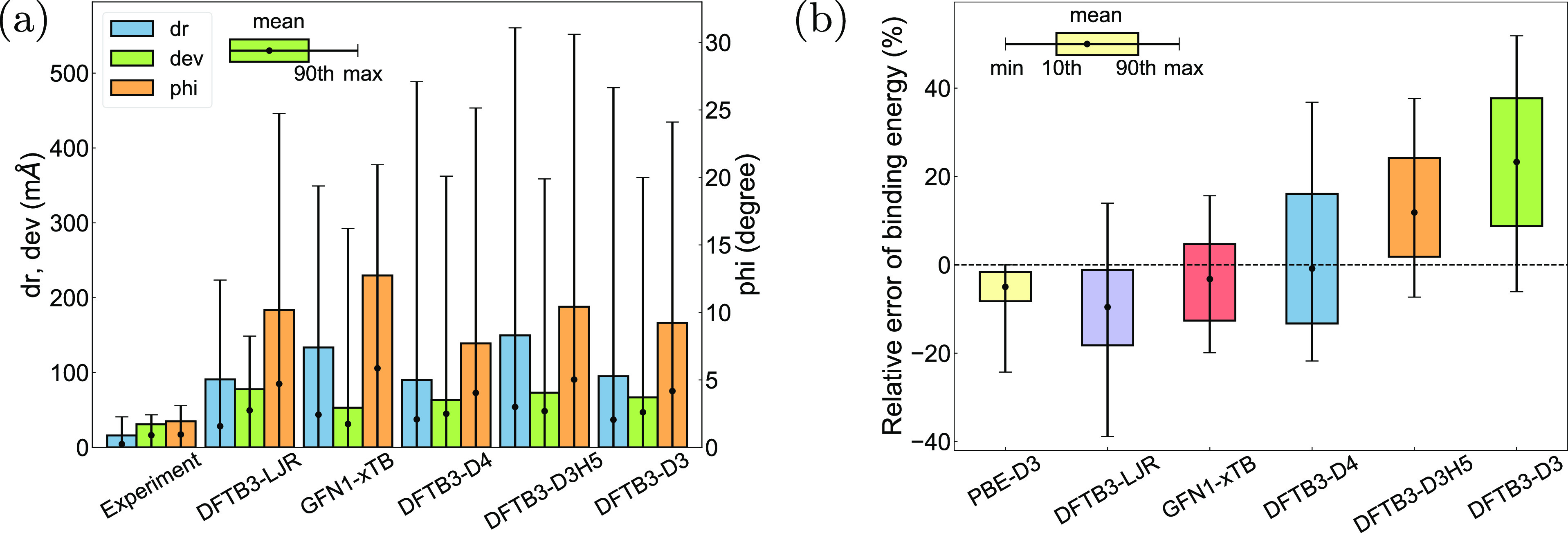
Benchmark of approximate DFT methods against r^2^SCAN-D3 reference for (a) intramolecular (“dev”) and intermolecular (displacement “dr” and deflection “phi”) geometries and (b) binding energy.

Figure 4 summarizes the accuracy of the optimized crystal geometries. As illustrated in Figure 4a, all methods underestimate the r^2^SCAN-D3 unit cell volume except for DFTB3-LJR, which was tuned to match the r^2^SCAN-D3 equilibrium unit cell volume and binding energy, as described in Section 2. In terms of the unit cell shape (Figures 4b and S10), the DFTB3 methods exhibit higher accuracy compared to the GFN1-xTB techniques, especially DFTB3-LJR and DFTB3-D4. Other structural parameters describing the intramolecular geometry and molecular arrangement inside the unit cell are given in Figure 5a. For the intermolecular arrangement (“dr” and “phi”), most DFTB3 methods demonstrate higher accuracy than their GFN1-xTB counterpart, whereas for the intramolecular geometry (“dev”), the trend is opposite.

The performance of approximate DFT methods in terms of the binding energy at relaxed geometries is shown in Figure 5b. Overall, errors in energies and volume correlate (Figure 4a), albeit with a larger magnitude of deviations from DFT-D. The most accurate method is GFN1-xTB; its energies are unbiased with respect to DFT-D but have a larger spread of deviations compared to the PBE-D3 reference. However, the relaxed geometries obtained by the GFN1-xTB method exhibit large deviations in the unit cell volume and shape (Figure 4), so that accurate binding energies may result from some error compensation. Further insights can be gained from Figure S13, where GFN1-xTB binding energies are calculated for the relaxed r^2^SCAN-D3 geometries, showing a systematic underestimation of the DFT-D reference energies by several percent.

To study the chemical space dependence of the performance of approximate DFT methods, the entire BMCOS1 data set was partitioned into several groups based on their chemical composition (Table S19). The group-specific benchmarks (Figure S14) show that the considered DFTB methods are more accurate for pure hydrocarbons, whereas the largest errors in geometry come from systems with sulfur, chlorine, cyano-group, and combinations of several light chemical elements (CNH(O) group in Table S19). In contrast, GFN1-xTB shows uniform performance (with large error dispersion) across the entire chemical space. In order to further identify the outliers in the DFTB3-LJR and GFN1-xTB methods, a comparative analysis against experimental data is presented in Figure S15. Similar to PBE-D3, systems with polar bonds (TCNQ family and hexafluorobenzene) are among the outliers for both methods, though the trend is less systematic. Other outliers are coronene for DFTB3-LJR and tetrathiafulvalene (TTF) for GFN1-xTB.

## 6 Conclusions

The diversity of organic semiconductors has grown tremendously in the last two decades, triggering the concomitant development of various modeling approaches. Except for several thoroughly studied systems, the majority of available experimental and calculated data contain large uncertainties due to the multiscale complexity of these materials. At the same time, no high-level theoretical methods exist to provide robust reference data for bulk organic semiconductors except for a few molecular crystals with a small enough number of atoms in the unit cell. The proposed BMCOS1 data set serves the purpose of testing modeling methods for the structural and electronic properties of organic semiconductors made up of relatively rigid π-conjugated molecules. Among the computationally feasible DFT-D methods considered, r^2^SCAN-D3 proves to be the most reliable, while PBE-D3 emerges as the most accurate method for predicting the unit cell volume within 1% accuracy, with the exception of molecules where PBE produces inaccurate atomic charges. When it comes to scalability, PBE-D3 continues to be the primary choice for crystals with hundreds of atoms in the unit cell. However, there is a possibility of overestimating the lengths of intermolecular contacts involving fluorine atoms. To address this issue in a computationally efficient manner, further investigation is required, including an assessment of the potential importance of range separation in dispersion interactions. In this study, all DFTB3- and xTB-based methods utilizing a universal parametrization exhibit reasonable accuracy, albeit with a consistent underestimation of the unit cell volume by several percentage points. However, this bias can be rectified by rescaling the parameters of the dispersion corrections, as demonstrated in the DFTB3-LJR method.

Altogether, the BMCOS1 database lays the foundation for the creation of various benchmark-type data sets for organic semiconductors, to include polymorphs, flexible molecules, polymers, metal–organic systems, and more. Our current data set not only provides a platform for designing and testing reduced atomistic quantum-mechanical models aiming to achieve DFT-D accuracy for molecular solids but also serves as a crucial reference and training set for assessing advanced interatomic potentials, including those generated through machine learning methodologies.^64^

## Supporting Information Available

Technical details of calculations and a snapshot of the database (core files) (ZIP)
(PDF)

## References

[ref1] Rivnay J.; Mannsfeld S. C. B.; Miller C. E.; Salleo A.; Toney M. F. Quantitative Determination of Organic Semiconductor Microstructure from the Molecular to Device Scale. Chem. Rev. 2012, 112, 5488–5519. 10.1021/cr3001109. 22877516

[ref2] Bhat V.; Callaway C.; Risko C. Computational Approaches for Organic Semiconductors: From Chemical and Physical Understanding to Predicting New Materials. Chem. Rev. 2023, 123, 7498–7547. 10.1021/acs.chemrev.2c00704. 37141497

[ref3] Oberhofer H.; Reuter K.; Blumberger J. Charge Transport in Molecular Materials: An Assessment of Computational Methods. Chem. Rev. 2017, 117, 10319–10357. 10.1021/acs.chemrev.7b00086. 28644623

[ref4] Davey R. J.; Schroeder S. L. M.; ter Horst J. H. . Nucleation of Organic Crystals—A Molecular Perspective. Angew. Chem., Int. Ed. 2013, 52, 2166–2179. 10.1002/anie.201204824. 23307268

[ref5] Zhang X.; Dong H.; Hu W. Organic Semiconductor Single Crystals for Electronics and Photonics. Adv. Mater. 2018, 30, 1801048 10.1002/adma.201801048. 30039629

[ref6] Jiang H.; Hu W. The Emergence of Organic Single Crystal Electronics. Angew. Chem., Int. Ed. 2020, 59, 1408–1428. 10.1002/anie.201814439. 30927312

[ref7] Wang C.; Dong H.; Jiang L.; Hu W. Organic semiconductor crystals. Chem. Soc. Rev. 2018, 47, 422–500. 10.1039/C7CS00490G. 29186226

[ref8] Chen J.; Zhang W.; Wang L.; Yu G. Recent Research Progress of Organic Small-Molecule Semiconductors with High Electron Mobilities. Adv. Mater. 2023, 35, 2210772 10.1002/adma.202210772. 36519670

[ref9] Zhugayevych A.; Mazaleva O.; Naumov A.; Tretiak S. Lowest-energy crystalline polymorphs of P3HT. J. Phys. Chem. C 2018, 122, 9141–9151. 10.1021/acs.jpcc.7b11271.

[ref10] Harrelson T. F.; Cheng Y. Q.; Li J.; Jacobs I. E.; Ramirez-Cuesta A. J.; Faller R.; Moule A. J. Identifying Atomic Scale Structure in Undoped/Doped Semicrystalline P3HT Using Inelastic Neutron Scattering. Macromol 2017, 50, 2424–2435. 10.1021/acs.macromol.6b02410.

[ref11] Kapaev R.; Zhugayevych A.; Ryazantsev S.; Aksyonov D.; Novichkov D.; Matveev P.; Stevenson K. Charge storage mechanisms of a pi-d conjugated polymer for advanced alkali-ion battery anodes. Chem. Sci. 2022, 13, 8161–8170. 10.1039/D2SC03127B. 35919425 PMC9278342

[ref12] Harrelson T.; Dantanarayana V.; Xie X.; Koshnick C.; Nai D.; Fair R.; Nunez S.; Thomas A.; Murrey T.; Hickner M.; Grey J.; Anthony J.; Gomez E.; Troisi A.; Faller R.; Moule A. Direct probe of the nuclear modes limiting charge mobility in molecular semiconductors. Mater. Horiz. 2019, 6, 182–191. 10.1039/C8MH01069B.

[ref13] Organic Materials Database (OMDB), https://omdb.mathub.io (accessed 04 28, 2023). .

[ref14] Borysov S.; Geilhufe R.; Balatsky A. Organic materials database: An open-access online database for data mining. PLoS One 2017, 12, e0171501 10.1371/journal.pone.0171501. 28182744 PMC5300202

[ref15] Organic Crystals in Electronic and Light-Oriented Technologies (OCELOT) database, https://oscar.as.uky.edu (accessed 04 28, 2023).

[ref16] Ai Q.; Bhat V.; Ryno S.; Jarolimek K.; Sornberger P.; Smith A.; Haley M.; Anthony J.; Risko C. OCELOT: An infrastructure for data-driven research to discover and design crystalline organic semiconductors. J. Chem. Phys. 2021, 154, 174705 10.1063/5.0048714. 34241085

[ref17] Yavuz I.; Lopez S. A.; Lin J. B.; Houk K. N. Quantitative prediction of morphology and electron transport in crystal and disordered organic semiconductors. J. Mater. Chem. C 2016, 4, 11238–11243. 10.1039/c6tc03823a.

[ref18] Reilly A. M.; Cooper R. I.; Adjiman C. S.; Bhattacharya S.; Boese A. D.; Brandenburg J. G.; Bygrave P. J.; Bylsma R.; Campbell J. E.; Car R.; et al. Report on the sixth blind test of organic crystal structure prediction methods. Acta Cryst. B 2016, 72, 439–459. 10.1107/s2052520616007447. PMC4971545 27484368

[ref19] Brandenburg J. G.; Grimme S. Organic crystal polymorphism: a benchmark for dispersion-corrected mean-field electronic structure methods. Acta Cryst. B 2016, 72, 502–513. 10.1107/S2052520616007885. 27484372

[ref20] Dolgonos G.; Hoja J.; Boese A. Revised values for the X23 benchmark set of molecular crystals. Phys. Chem. Chem. Phys. 2019, 21, 24333–24344. 10.1039/C9CP04488D. 31675024

[ref21] Control and Prediction of the Organic Solid State (CPOSS) database. http://www.chem.ucl.ac.uk/cposs (accessed 04 28, 2023).

[ref22] Price S. Control and prediction of the organic solid state: a challenge to theory and experiment <sup/>. Proc. R. Soc. A 2018, 474, 20180351 10.1098/rspa.2018.0351. 30333710 PMC6189584

[ref23] Sure R.; Grimme S. Comprehensive Benchmark of Association (Free) Energies of Realistic Host-Guest Complexes. J. Chem. Theory Comput. 2015, 11, 3785–3801. 10.1021/acs.jctc.5b00296. 26574460

[ref24] Sedlak R.; Janowski T.; Pitoňák M.; Řezáč J.; Pulay P.; Hobza P. Accuracy of Quantum Chemical Methods for Large Noncovalent Complexes. J. Chem. Theory Comput. 2013, 9, 3364–3374. 10.1021/ct400036b. 24098094 PMC3789125

[ref25] Schatschneider B.; Monaco S.; Liang J.; Tkatchenko A. High-Throughput Investigation of the Geometry and Electronic Structures of Gas-Phase and Crystalline Polycyclic Aromatic Hydrocarbons. J. Phys. Chem. C 2014, 118, 19964–19974. 10.1021/jp5064462.

[ref26] Grimme S.; Antony J.; Ehrlich S.; Krieg H. A consistent and accurate ab initio parametrization of density functional dispersion correction (DFT-D) for the 94 elements H-Pu. J. Chem. Phys. 2010, 132, 154104 10.1063/1.3382344. 20423165

[ref27] Ehlert S.; Huniar U.; Ning J.; Furness J.; Sun J.; Kaplan A.; Perdew J.; Brandenburg J. r2SCAN-D4: Dispersion corrected meta-generalized gradient approximation for general chemical applications. J. Chem. Phys. 2021, 154, 061101 10.1063/5.0041008. 33588552

[ref28] Caldeweyher E.; Ehlert S.; Hansen A.; Neugebauer H.; Spicher S.; Bannwarth C.; Grimme S. A generally applicable atomic-charge dependent London dispersion correction. J. Chem. Phys. 2019, 150, 154122 10.1063/1.5090222. 31005066

[ref29] Halaby S.; Martynowycz M. W.; Zhu Z.; Tretiak S.; Zhugayevych A.; Gonen T.; Seifrid M. Microcrystal Electron Diffraction for Molecular Design of Functional Non-Fullerene Acceptor Structures. Chem. Mater. 2021, 33, 966–977. 10.1021/acs.chemmater.0c04111. 36942096 PMC10024952

[ref30] Tukachev N. V.; Maslennikov D. R.; Sosorev A. Y.; Tretiak S.; Zhugayevych A. Ground state geometry and vibrations of polyphenylenevinylene oligomers. J. Phys. Chem. Lett. 2019, 10, 3232–3239. 10.1021/acs.jpclett.9b01200. 31141372

[ref31] Cui Q.; Elstner M. Density functional tight binding: values of semi-empirical methods in an ab initio era. Phys. Chem. Chem. Phys. 2014, 16, 14368–14377. 10.1039/C4CP00908H. 24850383 PMC4836871

[ref32] Elstner M.; Porezag D.; Jungnickel G.; Elsner J.; Haugk M.; Frauenheim T.; Suhai S.; Seifert G. Self-consistent-charge density-functional tight-binding method for simulations of complex materials properties. Phys. Rev. B 1998, 58, 7260–7268. 10.1103/PhysRevB.58.7260.

[ref33] Bannwarth C.; Caldeweyher E.; Ehlert S.; Hansen A.; Pracht P.; Seibert J.; Spicher S.; Grimme S. Extended tight-binding quantum chemistry methods. Wiley Interdiscip. Rev.: Comput. Mol. Sci. 2021, 11, e1493 10.1002/wcms.1493.

[ref34] Grimme S.; Bannwarth C.; Shushkov P. A Robust and Accurate Tight-Binding Quantum Chemical Method for Structures, Vibrational Frequencies, and Noncovalent Interactions of Large Molecular Systems Parametrized for All spd-Block Elements (Z. = 1–86). J. Chem. Theory Comput. 2017, 13, 1989–2009. 10.1021/acs.jctc.7b00118. 28418654

[ref35] Gaus M.; Cui Q.; Elstner M. DFTB3: Extension of the Self-Consistent-Charge Density-Functional Tight-Binding Method (SCC-DFTB). J. Chem. Theory Comput. 2011, 7, 931–948. 10.1021/ct100684s. PMC3509502 23204947

[ref36] Kubillus M.; Kubař T.; Gaus M.; Řezáč J.; Elstner M. Parameterization of the DFTB3Method for Br, Ca, Cl, F, I, K, and Na in Organic and Biological Systems. J. Chem. Theory Comput. 2015, 11, 332–342. 10.1021/ct5009137. 26889515

[ref37] Brandenburg J.; Grimme S. Accurate Modeling of Organic Molecular Crystals by Dispersion-Corrected Density Functional Tight Binding (DFTB). J. Phys. Chem. Lett. 2014, 5, 1785–1789. 10.1021/jz500755u. 26273854

[ref38] Mortazavi M.; Brandenburg J.; Maurer R.; Tkatchenko A. Structure and Stability of Molecular Crystals with Many-Body Dispersion-Inclusive Density Functional Tight Binding. J. Phys. Chem. Lett. 2018, 9, 399–405. 10.1021/acs.jpclett.7b03234. 29298075

[ref39] Dolgonos G.; Boese A. Adjusting dispersion parameters for the density-functional tight-binding description of molecular crystals. Chem. Phys. Lett. 2019, 718, 7–11. 10.1016/j.cplett.2019.01.027.

[ref40] Bannwarth C.; Ehlert S.; Grimme S. GFN2-xTB - An Accurate and Broadly Parametrized Self-Consistent Tight-Binding Quantum Chemical Method with Multipole Electrostatics and Density-Dependent Dispersion Contributions. J. Chem. Theory Comput. 2019, 15, 1652–1671. 10.1021/acs.jctc.8b01176. 30741547

[ref41] Kresse G.; Furthmuller J. Efficient Iterative Schemes for Ab Initio Total-Energy Calculations Using a Plane-Wave Basis Set. Phys. Rev. B 1996, 54, 11169–11186. 10.1103/PhysRevB.54.11169. 9984901

[ref42] Sewell T.; Menikoff R.; Bedrov D.; Smith G. A molecular dynamics simulation study of elastic properties of HMX. J. Chem. Phys. 2003, 119, 7417–7426. 10.1063/1.1599273.

[ref43] Frisch M. J.; Gaussian 16, Revision C.01; Gaussian Inc, Wallingford CT, 2016.

[ref44] Hourahine B.; Aradi B.; Blum V.; Bonafe F.; Buccheri A.; Camacho C.; Cevallos C.; Deshaye M.; Dumitrică T.; Dominguez A.; Ehlert S.; Elstner M.; van der Heide T.; Hermann J.; Irle S.; Kranz J.; Kohler C.; Kowalczyk T.; Kubař T.; Lee I.; Lutsker V.; Maurer R.; Min S.; Mitchell I.; Negre C.; Niehaus T.; Niklasson A.; Page A.; Pecchia A.; Penazzi G.; Persson M.; Řezáč J.; Sanchez C.; Sternberg M.; Stohr M.; Stuckenberg F.; Tkatchenko A.; Yu V.; Frauenheim T. DFTB+, a software package for efficient approximate density functional theory based atomistic simulations. J. Chem. Phys. 2020, 152, 124101 10.1063/1.5143190. 32241125

[ref45] Grimme S.; Ehrlich S.; Goerigk L. Effect of the damping function in dispersion corrected density functional theory. J. Comput. Chem. 2011, 32, 1456–1465. 10.1002/jcc.21759. 21370243

[ref46] Řezáč J.; Hobza P. Advanced Corrections of Hydrogen Bonding and Dispersion for Semiempirical Quantum Mechanical Methods. J. Chem. Theory Comput. 2012, 8, 141–151. 10.1021/ct200751e. 26592877

[ref47] Zhechkov L.; Heine T.; Patchkovskii S.; Seifert G.; Duarte H. An Efficient a Posteriori Treatment for Dispersion Interaction in Density-Functional-Based Tight Binding. J. Chem. Theory Comput. 2005, 1, 841–847. 10.1021/ct050065y. 26641900

[ref48] Rappe A.; Casewit C.; Colwell K.; Goddard W.; Skiff W. UFF, a full periodic table force field for molecular mechanics and molecular dynamics simulations. J. Am. Chem. Soc. 1992, 114, 10024–10035. 10.1021/ja00051a040.

[ref49] Kearsley S. K. On the orthogonal transformation used for structural comparisons. Acta Cryst. A 1989, 45, 208–210. 10.1107/S0108767388010128.

[ref50] Honig S.; Lemmen C.; Rarey M. Small molecule superposition: A comprehensive overview on pose scoring of the latest methods. Wiley Interdiscip. Rev.: Comput. Mol. Sci. 2023, 13, e1640 10.1002/wcms.1640.

[ref51] Gray M.; Herbert J. Origins of Offset-Stacking in Porous Frameworks. J. Phys. Chem. C 2023, 127, 2675–2686. 10.1021/acs.jpcc.2c08413.

[ref52] Zhugayevych A.; Postupna O.; Wang H. L.; Tretiak S. Modification of optoelectronic properties of conjugated oligomers due to donor/acceptor functionalization: DFT study. Chem. Phys. 2016, 481, 133–143. 10.1016/j.chemphys.2016.09.009.

[ref53] Landrum G. A.; Hoffmann R. Secondary bonding between chalcogens or pnicogens and halogens. Angew. Chem., Int. Ed. 1998, 37, 1887–1890. 10.1002/(SICI)1521-3773(19980803)37:13/14<1887::AID-ANIE1887>3.0.CO;2-0.

[ref54] Zhugayevych A.; Lubchenko V. Electronic structure and the glass transition in pnictide and chalcogenide semiconductor alloys. I. The formation of the *pp*σ-network. J. Chem. Phys. 2010, 133, 234503 10.1063/1.3511707. 21186871

[ref55] Poriel C.; Rault-Berthelot J. Designing Host Materials for the Emissive Layer of Single-Layer Phosphorescent Organic Light-Emitting Diodes: Toward Simplified Organic Devices. Adv. Funct. Mater. 2021, 31, 2010547 10.1002/adfm.202010547.

[ref56] Schober C.; Reuter K.; Oberhofer H. Virtual Screening for High Carrier Mobility in Organic Semiconductors. J. Phys. Chem. Lett. 2016, 7, 3973–3977. 10.1021/acs.jpclett.6b01657. 27661442

[ref57] Xie Y.; Fujimoto T.; Dalgleish S.; Shuku Y.; Matsushita M.; Awaga K. Synthesis, optical properties and charge transport characteristics of a series of novel thiophene-fused phenazine derivatives. J. Mater. Chem. C 2013, 1, 3467–3481. 10.1039/c3tc30346b.

[ref58] Ko H.; DiStasio R.; Santra B.; Car R. Thermal expansion in dispersion-bound molecular crystals. Phys. Rev. Mater. 2018, 2, 055603 10.1103/PhysRevMaterials.2.055603.

[ref59] van der Lee A.; Dumitrescu D. Thermal expansion properties of organic crystals: a CSD study. Chem. Sci. 2021, 12, 8537–8547. 10.1039/D1SC01076J. 34221335 PMC8221191

[ref60] Hoja J.; Reilly A. M.; Tkatchenko A. First-principles modeling of molecular crystals: structures and stabilities, temperature and pressure. Wiley Interdiscip. Rev.: Comput. Mol. Sci. 2016, 7, e1294 10.1002/wcms.1294.

[ref61] Thalladi V.; Weiss H.; Blaser D.; Boese R.; Nangia A.; Desiraju G. R. C–H···F Interactions in the Crystal Structures of Some Fluorobenzenes. J. Am. Chem. Soc. 1998, 120, 8702–8710. 10.1021/ja981198e.

[ref62] Dupuy N.; Casula M. Fate of the open-shell singlet ground state in the experimentally accessible acenes: A quantum Monte Carlo study. J. Chem. Phys. 2018, 148, 134112 10.1063/1.5016494. 29626884

[ref63] Mouhat F.; Coudert F. Necessary and sufficient elastic stability conditions in various crystal systems. Phys. Rev. B 2014, 90, 224104 10.1103/PhysRevB.90.224104.

[ref64] Anstine D.; Isayev O. Machine Learning Interatomic Potentials and Long-Range Physics. J. Phys. Chem. A 2023, 127, 2417–2431. 10.1021/acs.jpca.2c06778. 36802360 PMC10041642

