# Supplementary material for: Benchmark Data Set of Crystalline Organic Semiconductors
Source: J Chem Theory Comput. 2023 Nov 16;19(22):8481–90. doi: 10.1021/acs.jctc.3c00861 (PMC10688188; doi:10.1021/acs.jctc.3c00861)
Supplement: Supplementary file 2 — ct3c00861_si_002.pdf [file ct3c00861_si_002.pdf]

Supporting Information for Benchmark dataset of crystalline organic semiconductors

Andriy Zhugayevych,*† Wenbo Sun,‡ Tammo van der Heide,‡ Carlos R. Lien-Medrano,‡ Thomas Frauenheim,‡ Sergei Tretiak*¶

†*Max Planck Institute for Polymer Research, Ackermannweg 10, 55128 Mainz, Germany*

‡*Bremen Center for Computational Materials Science, Am Fallturm 1, 28359 Bremen, Germany*

¶*Los Alamos National Laboratory, NM 87545, United States*

*E-mail: andriy.zhugayevych@mpip-mainz.mpg.de; serg@lanl.gov

November 2, 2023

S1 Computational methodology: abbreviations and notations . . . S1
S2 List of molecules . . . S3
S3 DFTB parameters . . . S5
S4 Supporting model systems . . . S6
S5 Additional figures and tables copied from the project webpage . . . S7
S6 Additional figures and tables for approximate DFT methods . . . S22

S1 Computational methodology: abbreviations and notations

a3p	Ahlrichs triple-ζ basis Def2-TZVP	MD	Molecular Dynamics
AO	Atomic Orbital	MO	Molecular Orbital
BMCOS	Benchmark Dataset of Crystalline Organic Semiconductors	MWD	Mass-Weighted Displacement
CIF	Crystallographic Information File	PAW	Projector Augmented Wave
CSD	Cambridge Structural Database	PES	Potential Energy Surface
EoS	Equation of State	p2p	Pople double-ζ polarized basis 6-31G*
DFT	Density Functional Theory	RMS	Root-Mean Square
DFT-D	Dispersion-corrected DFT	SCF	Self-Consistent Field
DFTB	Density Functional based Tight Binding	TB	Tight Binding
HOMO	Highest Occupied Molecular Orbital	ZPE	Zero-Point Energy
LUMO	Lowest Unoccupied Molecular Orbital		

BMCOS dataname notations (alphabetical order) [URL]

To minimize use of special symbols we denote Ao=Å, deg=degree (angle), iX=X^(-1), X/mol=X per molecule, dX=deviation of X in % except for angles.

- `[a,b,c,alpha,beta,gamma]` (Ao,deg) unit cell parameters
- `[dr,phi,dev]` (mAo,deg,mAo) displacement, deflection, and RMS deviation of single-molecule geometry without hydrogens (molecules are superimposed individually by MolMod/Superimpose, the result is RMS-averaged over all symmetry-unique molecules)
- `al=alpha` (ppm/K) thermal expansion coefficient
- `conf` conformation or configuration
- `dbet` (deg) absolute deviation of monoclinic angle
- `dHOMO,dLUMO` (eV) energy gap between HOMO/LUMO and next HOMO/LUMO
- `dlen` (%) deviation of length of translation vectors (three-dimensional vector)
- `dSh` (%) deviation of shape of unit cell, the same as `dTv` but with volume rescaled to unity
- `dTv` (%) RMS deviation of translation vectors (Frobenius norm of the unit cell matrix divided by square root of the number of translation vectors) divided by cubic root of the unit cell volume (the compared unit cell is rotated to minimize this RMS deviation, the code is BasicTools/SuperimposeM)
- `Eb` (eV/mol) binding energy
- `el` list of chemical elements
- `EM,EM1` (GPa) elastic matrix (elasticity tensor in Voigt notation) and its lowest eigenvalue
- `freq1` (meV) lowest vibrational frequency
- `gap` (eV) HOMO-LUMO gap
- `id=sys` BMCOS system identifier, usually a common name of a molecule
- `K,GR,GV` (GPa) bulk and shear moduli calculated from `EM` by MolMod/exam_vas/PrintFreq [Sewell03]
- `K0,K0'` (GPa,1) bulk modulus and its derivative calculated by Murnaghan equation of state fit, the code is MolMod/EOSfit
- `mult=m` number of primitive cells in Bravais unit cell
- `na` number of atoms in unit cell
- `na1,nas,nu=Z` number of atoms in the smallest repeating unit, list of number of atoms in molecules from this unit, and number of such units
- `name` IUPAC-consistent chemical name of a molecule
- `nb` number of electronic bands (NBANDS)
- `nd` number of data points
- `ng` number of elements in `SG`
- `nkp` number of k-points
- `nm` number of molecules in unit cell (usually Bravais unit cell)
- `no` number of symmetry-unique molecules, i.e. number of orbits in `orbs`
- `nopt` number of geometry relaxation iterations
- `nscf` number of SCF iterations
- `nstab=ns` number of elements in point symmetry group (stabilizer) of molecules in crystal
- `orbs` symmetry-generated orbits of molecules in unit cell as list of lists of molecule indexes
- `PG` point group
- `pubchem` PubChem identifier
- `SG` symmetry group or space group if combined with `PG`
- `T` (K) temperature
- `td` (days) wall-clock computing time in days
- `Up2=|G|,maxG` (meV/Ao) RMS norm and maximum absolute force on atoms
- `V` (Ao^3) unit cell volume
- `V0` (Ao^3) volume per atom extrapolated to 0K
- `V1` (Ao^3) volume per atom

S2 List of molecules

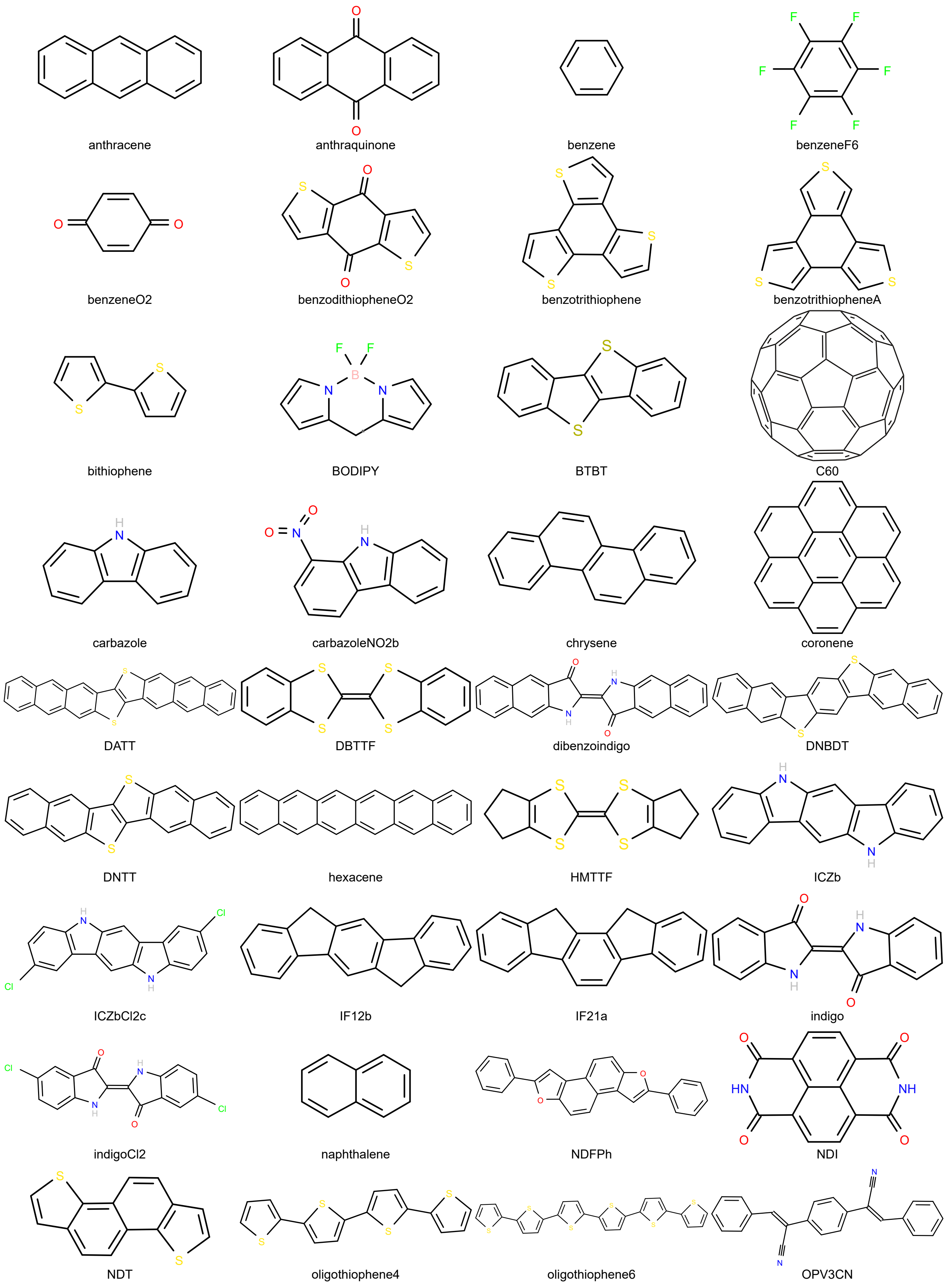

Figure S1: List of molecules, part 1: A-O.

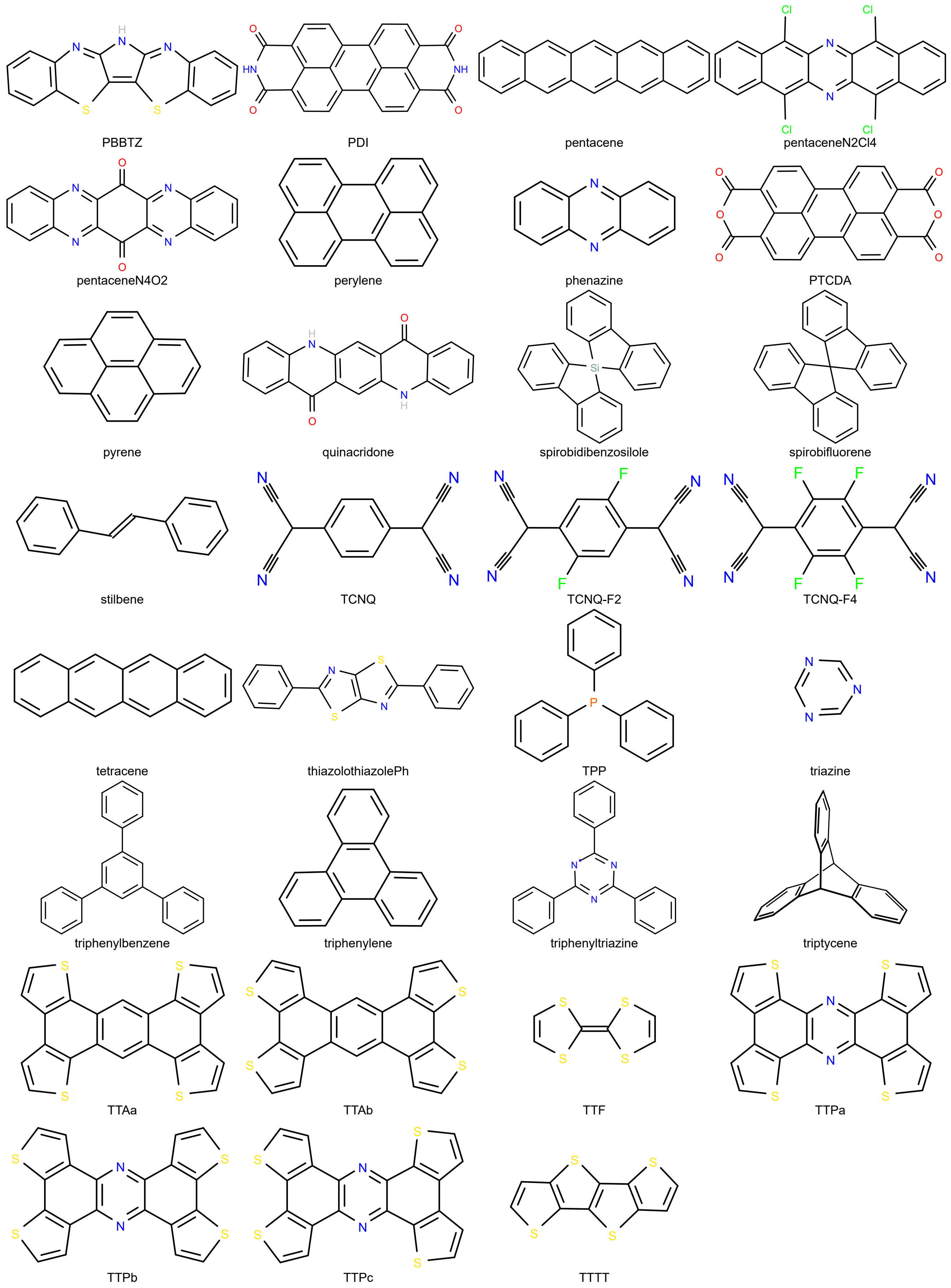

Figure S2: List of molecules, part 2: P-Z.

S3 DFTB parameters

Table S1: Lennard–Jones dispersion parameters used in DFTB3-LJ method.

elements	distance x_I^a [Å]	D_I^c [kcal/mol]
H	2.886	0.044
C	3.851	0.105
N	3.660	0.069
O	3.500	0.060
F	3.364	0.050
P	4.147	0.305
S	4.035	0.274
Cl	3.947	0.227

Table S2: Lennard–Jones dispersion parameters used in the DFTB3-LJR method. The UFF distances and energies of Table S1 have been rescaled by 0.9608 and 0.7970 respectively, according to the average signed deviation of Fig. S11 and Fig. S12.

elements	distance x_I^a [Å]	D_I^c [kcal/mol]
H	2.7728688	0.035068
C	3.7000408	0.083685
N	3.5165280	0.054993
O	3.3628000	0.047820
F	3.2321312	0.039850
P	3.9844376	0.243085
S	3.8768280	0.218378
Cl	3.7922776	0.180919

Table S3: Becke–Johnson damping parameters used in the DFTB3-D3(BJ) method.

parameters	s_6	s_8	a_1	a_2 [a_0]
3ob-3-1	1.0	3.209	0.746	4.191

Table S4: Zero-damping parameters used in the DFTB3-D3H5 method.

parameters	s_6	s_8	$s_{r,6}$	α_6
3ob-3-1	1.0	0.49	1.25	29.61

Table S5: H5 hydrogen correction parameters used in the DFTB3-D3H5 method.

parameters	s_r	s_w	k_OH	k_NH	k_SH
3ob-3-1	0.714	0.25	0.06	0.18	0.21

Table S6: Becke–Johnson damping parameters used in the DFTB3-D4(BJ) method.

parameters	s_6	s_8	s_9	a_1	a_2 [a_0]
3ob-3-1	1.0	0.6635015	1.0	0.5523240	4.3537076

S4 Supporting model systems

Table S7: Dependence of the electronic band energy of a half-filled dimerized chain on boundary conditions (TB model of trans-polyacetylene and its annulenes). Here t and t' are transfer integrals of primary ("double") and secondary ("single") bonds. In this model the monomer is a pair of sites connected by the primary bond. These monomers are interconnected by weak secondary bonds. We divide the total energy by number of monomers and subtract the electronic energy of the monomer, $-2t$, so that the result is the band contribution to the binding energy of monomers in a chain. Here E' is the complementary complete elliptic integral. Notice that in terms of electronic levels, circle of N monomers is equivalent to the infinite chain on a finite Γ-centered grid with N k-points. In this context, Γ-point calculations introduce an "extra-bond" artifact, $2t'$. In 2 k-points calculations the secondary bonding energy is missing, which is acceptable for molecular crystals without strong donor-acceptor intermolecular interactions.

Boundary conditions	Binding energy	Expanded in t'/t
infinite chain	$\frac{4}{\pi}(t+t')\mathrm{E}'\left(\frac{t-t'}{t+t'}\right) - 2t$	$\frac{t'^2}{2t} + \frac{t'^4}{32t^3}$
circle of 4 monomers	$\sqrt{t^2+t'^2} - t$	$\frac{t'^2}{2t} - \frac{t'^4}{8t^3}$
circle of 3 monomers	$\frac{4}{3}\sqrt{t^2-tt'+t'^2} - \frac{4}{3}t + \frac{2}{3}t'$	$\frac{t'^2}{2t} + \frac{t'^3}{4t^2} + \frac{t'^4}{32t^3}$
circle of 2 monomers	0	0
circle of 1 monomer	$2t'$	$2t'$
dimer	$\sqrt{4t^2+t'^2} - 2t$	$\frac{t'^2}{4t} - \frac{t'^4}{64t^3}$

S5 Additional figures and tables copied from the project webpage

Table S8: Datasheets: core data [URL].

sys	conf	na1	nm	na	el	SG1	SG	pubchem	name
benzene	cryst	12	4	48		6/mmm	Pbca	241	benzene
naphthalene	cryst	18	2	36		mmm	P21/c	931	naphthalene
anthracene	cryst	24	2	48		mmm	P21/c	8418	anthracene
tetracene	cryst	30	2	60		mmm	P-1	7080	tetracene
pentacene	cryst	36	2	72		mmm	P-1	8671	pentacene
hexacene	cryst	42	2	84		mmm	P-1	123044	hexacene
chrysene	cryst	30	4	120		2/m	C2/c	9171	chrysene
pyrene	cryst	26	4	104		mmm	P21/c	31423	pyrene
perylene	cryst	32	2	64		mmm	P21/c	9142	perylene
IF12b	cryst	34	2	68		2/m	P21/c	15559295	6,12-dihydroindeno[1,2-b]fluorene
IF21a	cryst	34	4	136		mm2	P21/c	15559296	11,12-dihydroindeno[2,1-a]fluorene
triphenylene	cryst	30	4	120		-62m	P212121	9170	triphenylene
coronene	cryst	36	2	72		6/mmm	P21/c	9115	coronene
stilbene	cryst	26	4	104		2/m	P21/c	638088	stilbene
triazine	cryst	9	4	36	N	-62m	C2/c	9262	s-triazine
phenazine	cryst	22	2	44	N	mmm	P21/c	4757	phenazine
carbazole	cryst	22	4	88	N	mm2	Pnma	6854	carbazole
ICZb	cryst	32	2	64	N	2/m	P21/c	114764	indolo[3,2-b]carbazole
TCNQ	cryst	20	4	80	N	mmm	C2/c	73697	tetracyanoquinodimethane
TCNQ-F2	cryst	20	2	40	NF	2/m	C2/m	5255840	2,5-difluoro-7,7,8,8-tetracyanoquinodimethane
TCNQ-F4	cryst	20	4	80	NF	mmm	Pbca	2733307	2,3,5,6-tetrafluoro-7,7,8,8-tetracyanoquinodimethane
ICZbCl2c	cryst	32	2	64	NCl	2/m	P21/c	66722443	2,8-dichloroindolo[3,2-b]carbazole
pentaceneN2Cl4	cryst	34	2	68	NCl	mmm	P21/c	91402528	5,7,12,14-tetrachloro-6,13-diazapentacene
TTTT	cryst	18	2	36	S	2/m	P21/c	14383681	thieno[3,2-b]thieno[2',3':4,5]thieno[2,3-d]thiophene
BTBT	cryst	24	2	48	S	2/m	P21/c	136056	[1]Benzothieno[3,2-b][1]benzothiophene
DNTT	cryst	36	2	72	S	2/m	P21	51050187	dinaphtho[2,3-b:2',3'-f]thieno[3,2-b]thiophene
DATT	cryst	48	2	96	S	2/m	P21	53308489	dianthra[2,3-b:2',3'-f]thieno[3,2-b]thiophene
DNBDT	cryst	42	2	84	S	2/m	P21/c	57913385	dinaphtho[2,3-d:2',3'-d']benzo[1,2-b:4,5-b']dithiophene
NDT	cryst	24	4	96	S	2/m	P21/c	58434773	naphtho[1,2-b:5,6-b']dithiophene
bithiophene	cryst	16	2	32	S	2/m	P21/c	68120	bithiophene
oligothiophene4	cryst	30	2	60	S	2/m	P21/c	86618470	quaterthiophene
oligothiophene6	cryst	44	2	88	S	2/m	P21/c	11340899	sexithiophene
benzotrithiophene	cryst	21	4	84	S	-6	P21/c	44225674	benzo[1,2-b:3,4-b':5,6-b'']trithiophene
benzotrithiopheneA	cryst	21	4	84	S	-62m	P21	2762442	benzo[1,2-c:3,4-c':5,6-c'']trithiophene
TTF	cryst	14	2	28	S	mmm	P21/c	99451	tetrathiafulvalene
HMTTF	cryst	28	4	112	S	mmm	P21/c	625027	hexamethylene-tetrathiafulvalene
DBTTF	cryst	26	2	52	S	mmm	P21/c	141136	dibenzotetrathiafulvalene
benzeneF6	cryst	12	6	72	F	6/mmm	P21/c	9805	hexafluorobenzene
benzeneO2	cryst	12	2	24	O	mmm	P21/c	4650	benzoquinone
anthraquinone	cryst	24	2	48	O	mmm	P21/c	6780	anthraquinone
PTCDA	cryst	38	2	76	O	mmm	P21/c	67191	perylenetetracarboxylic dianhydride
NDFPh	cryst	44	2	88	O	2/m	P21/c	89963552	naphtho[2,1-b:6,5-b']difuran
NDI	cryst	26	2	52	NO	mmm	P-1	79771	naphthalene-1,8:4,5-tetracarboxydiimide
PDI	cryst	40	2	80	NO	mmm	P21/c	66475	perylenediimide
quinacridone	cryst	36	2	72	NO	2/m	P21/c	13976	quinacridone
pentaceneN4O2	cryst	32	2	64	NO	mmm	P21/c	101507809	quinoxalino[2,3-b]phenazine-6,13-dione
indigo	cryst	30	2	60	NO	2/m	P21/c	10215	indigo
indigoCl2	cryst	30	2	60	NOCl	2/m	P21/c	136653217	5,5'-dichloroindigo
dibenzoindigo	cryst	42	2	84	NO	2/m	P21/c	0	dibenzoindigo
carbazoleNO2b	cryst	24	4	96	NO	m	P21/c	96730	1-nitrocarbazole
TTAa	cryst	36	2	72	S	mmm	P21/c	90727247	1,2:4,3:5,6:8,7-tetra(epithioetheno)anthracene
TTAb	cryst	36	2	72	S	mmm	P21/c	101860588	3,4,10,11-tetrathiatetracyclopenta[a,c,h,j]anthracene
TTPa	cryst	34	2	68	NS	mmm	P21/c	101566660	1,2:4,3:5,6:8,7-tetra(epithioetheno)phenazine
TTPb	cryst	34	4	136	NS	mmm	P21/c	102314304	3,4,10,11-tetrathiatetracyclopenta[a,c,h,j]phenazine
TTPc	cryst	34	2	68	NS	mm2	P21	0	m-tetrathienophenazine
PBBTZ	cryst	30	4	120	NS	mm2	P21/c	91574055	6h-pyrrolo[3,2-b:4,5-b']bis[1,4]benzothiazine
thiazolothiazolePh	cryst	30	2	60	NS	2/m	P21/c	240920	2,5-diphenyl(1,3)thiazolo(5,4-d)(1,3)thiazole
benzodithiopheneO2	cryst	18	2	36	OS	2/m	P21/c	288478	benzo[1,2-b:4,5-b']dithiophene-4,8-dione
C60	cryst	60	4	240		m-5	Pa-3	123591	buckminsterfullerene
triptycene	cryst	34	4	136		-62m	P212121	92764	triptycene
BODIPY	cryst	21	8	168	NFB	mm2	C2/c	25058173	4,4-difluoro-4-bora-3a,4a-diaza-s-indacene
OPV3CN	cryst	42	4	168	N	2/m	Pbca	54520030	1,4-bis(1-cyano-2-phenylethenyl)benzene
spirobifluorene	cryst	41	4	164		-42m	P21/c	135975	9,9'-spirobi[9H-fluorene]
spirobidibenzosilole	cryst	41	4	164	Si	-42m	P41212	135976	5,5'-spirobi(dibenzosilole)
triphenyltriazine	cryst	39	4	156	N	-62m	P21/c	10305	triphenyl-1,3,5-triazine
triphenylbenzene	cryst	42	4	168		32	Pna21	11930	1,3,5-triphenylbenzene
TPP	cryst	34	4	136	P	3	P21/c	11776	triphenylphosphine

Table S9: Datasheets: experimental data [URL].

sys	conf	T	al	nd	Tmin	Tmax	V1vsT
benzene	cryst	100	353	11	15	270	V0=9.51(7)
naphthalene	cryst	100	195	22	5	295	V0=9.378(19)
anthracene	cryst	94	179	22	90	295	V0=9.336(13)
tetracene	cryst	175	142	4	175	295	V0=9.31(11)
pentacene	cryst	180	183	16	90	498	V0=9.10(5)
hexacene	cryst	123	123	3	123	295	alpha(300K)=123
chrysene	cryst	125	184	3	125	295	V0=9.262(7)
pyrene	cryst	93	172	11	90	295	V0=9.610(16)
perylene	cryst	200	104	3	130	295	V0=9.192(2)
IF12b	cryst	296					OJONIL
IF21a	cryst	100					OJICOA
triphenylene	cryst	123	116	6	123	295	V0=9.33(4)
coronene	cryst	100	139	11	100	296	V0=9.534(16)
stilbene	cryst	90	194	20	90	373	V0=9.36(2)
triazine	cryst	5	138	2	5	150	alpha(300K)=138
phenazine	cryst	295	173	6	80	296	V0=9.65(4)
carbazole	cryst	100	179	6	100	295	V0=9.16(2)
ICZb	cryst	293					VARMUY
TCNQ	cryst	180	117	10	100	295	V0=12.31(3)
TCNQ-F2	cryst	180	184	5	100	295	V0=12.32(8)
TCNQ-F4	cryst	180	198	5	100	295	V0=13.181(19)
ICZbCl2c	cryst	113					VARNAF
pentaceneN2Cl4	cryst	150					UTEFEF
TTTT	cryst	295					JOTVAP
BTBT	cryst	100	89	5	90	293	V0=10.91(2)
DNTT	cryst	293					NICLAN
DATT	cryst	293					AVIBEN
DNBDT	cryst	298					MOGQAC
NDT	cryst	293					OKUPEQ
bithiophene	cryst	133	235	2	133	173	alpha(300K)=235
oligothiophene4	cryst	295	154	4	150	295	alpha(300K)=154
oligothiophene6	cryst	295					ZAQZUM
benzotrithiophene	cryst	293					ERIKIZ
benzotrithiopheneA	cryst	120					KISFEY
TTF	cryst	290	208	5	98	295	V0=13.53(4)
HMTTF	cryst	296					KIVDAV
DBTTF	cryst	123	148	3	123	295	V0=11.66(3)
benzeneF6	cryst	106	411	9	106	268	V0=11.83(9)
benzeneO2	cryst	173	313	4	113	298	V0=10.31(10)
anthraquinone	cryst	163	136	25	103	298	V0=9.62(4)
PTCDA	cryst	223					SUWMIG
NDFPh	cryst	296					CAWSAW
NDI	cryst	150	69	2	150	293	alpha(300K)=69
PDI	cryst	263		4	263	295	
quinacridone	cryst	293	187	2	223	293	alpha(300K)=187
pentaceneN4O2	cryst	296					WUPYIQ
indigo	cryst	210		3	210	295	
indigoCl2	cryst	100					MOGSEI
dibenzoindigo	cryst	110					noCCDC
carbazoleNO2b	cryst	100					RITMAK
TTAa	cryst	100					VOBYAN
TTAb	cryst	100					VOBYER
TTPa	cryst	173					SESSOA
TTPb	cryst	173					SESTAN
TTPc	cryst	298					SESSUG
PBBTZ	cryst	113					MOJFEX
thiazolothiazolePh	cryst	293	170	2	100	293	alpha(300K)=170
benzodithiopheneO2	cryst	296					ZAPZAT
C60	cryst	5	83	13	5	298	V0=11.478(21)
triptycene	cryst	100	117	5	100	295	V0=9.83(5)
BODIPY	cryst	213	234	3	173	296	alpha(300K)=234
OPV3CN	cryst	173	186	2	173	293	alpha(300K)=186
spirobifluorene	cryst	293	162	3	115	295	alpha(300K)=162
spirobidibenzosilole	cryst	100					SIBMUN
triphenyltriazine	cryst	123	164	4	100	295	V0=9.37(4)
triphenylbenzene	cryst	100	165	5	100	296	V0=9.59(4)
TPP	cryst	90	192	10	90	299	V0=10.11(4)

Table S10: Comparison wrt experiment for V1 extrapolated to 0K [URL].

system	na1	no	ns	SG	V1	R2S	PBE	PBE*
anthracene	24	1	2	P21/c	9.34	-2.0	0.4	
anthraquinone	24	1	2	P21/c	9.62	-2.6	0.5	
benzene	12	1	2	Pbca	9.51	-2.7	0.4	
benzeneF6	12	2	1	P21/n	11.83	0.8	12.1	(excluded)
benzeneO2	12	1	2	P21/c	10.31	-1.4	3.7	(excluded)
BTBT	24	1	2	P21/c	10.91	-1.5	0.0	
C60	60	1	6	Pa-3	11.48	0.5	1.9	
carbazole	22	1	2	Pnma	9.16	-1.9	0.6	
chrysene	30	1	2	C12/n1	9.26	-0.9	1.3	
coronene	36	1	2	P21/n	9.53	-1.0	0.5	
DBTTF	26	1	2	P21/c	11.66	-0.6	0.2	
naphthalene	18	1	2	P21/c	9.38	-2.5	0.4	
pentacene	36	2	2	P-1	9.10	-1.3	0.8	
perylene	32	1	2	P21/c	9.19	-2.3	-0.3	
phenazine	22	1	2	P21/n	9.65	-1.6	1.0	
pyrene	26	1	1	P21/a	9.61	-1.8	-0.1	
spirobifluorene	41	1	1	P21/c	10.05	-1.4	0.5	
stilbene	26	2	2	P21/a	9.36	-2.1	0.1	
TCNQ	20	1	2	C2/c	12.31	-1.8	0.9	
TCNQ-F2	20	1	4	C2/m	12.32	-0.5	4.2	(excluded)
TCNQ-F4	20	1	2	Pbca	13.18	-0.4	5.5	(excluded)
tetracene	30	2	2	P-1	9.31	-2.3	-0.1	
TPP	34	1	1	P21/c	10.11	-2.1	-0.4	
triphenylbenzene	42	1	1	Pna21	9.59	-2.7	-0.4	
triphenylene	30	1	1	P212121	9.33	-2.9	-0.8	
triphenyltriazine	39	1	1	P21/c	9.37	-2.0	0.6	
triptycene	34	1	1	P212121	9.83	-2.7	-0.7	
TTF	14	1	2	P21/c	13.53	-0.1	-0.2	
median						-1.8	0.4	0.4
mean						-1.6	1.2	0.3
standard dev						1.0	2.6	0.6
lower decile						-2.7	-0.4	-0.5
upper decile						-0.1	4.1	1.1
min						-2.9	-0.8	-0.8
max						0.8	12.1	1.9

Table S11: Comparison experiment wrt R2SCAN-D3paw900 [URL].

system	na1	no	ns	SG	V1	dV1	dSh	dTv	da	db	dc	dalp	dbet	dgam	dr	phi	dev
str	#	#	#	str	Ao3	%	%	%	%	%	%	deg	deg	deg	mAo	deg	mAo
median						4.2	0.8	1.7	1.1	1.3	1.3	0.0	0.1	0.0	0	0.8	13
mean						4.1	1.0	1.9	1.2	1.5	1.4	0.0	0.1	-0.0	4	1.0	16
standard dev						1.8	0.6	0.9	0.9	1.2	1.0	0.2	0.8	0.0	9	0.7	10
lower decile						2.0	0.3	0.9	0.5	0.2	0.4	0.0	-0.7	0.0	0	0.3	7
upper decile						6.6	1.8	3.2	2.2	3.1	2.5	0.0	0.8	0.0	17	2.0	31
min						-0.1	0.0	0.0	-0.6	-0.8	-1.1	-0.9	-2.6	-0.3	0	0.0	3
max						8.1	2.7	4.6	4.5	4.4	5.3	1.1	2.0	0.1	41	3.1	44
anthracene	24	1	2	P21/c	9.15	3.9	0.4	1.5	1.3	0.9	1.8	0.0	0.3	0.0	0	1.0	9
anthraquinone	24	1	2	P21/c	9.37	5.0	0.6	1.9	1.0	2.6	1.4	0.0	0.1	0.0	0	1.1	9
benzene	12	1	2	Pbca	9.25	7.0	0.8	2.5	1.2	2.8	2.9	0.0	0.0	0.0	0	1.3	3
benzeneF6	12	2	1	P21/n	11.93	4.7	1.3	2.0	0.5	3.4	0.8	0.0	0.2	0.0	0	1.5	10
benzeneO2	12	1	2	P21/c	10.17	7.7	2.3	3.7	1.7	1.1	5.3	0.0	2.0	0.0	0	2.6	9
benzodithiopheneO2	18	1	2	P21/n	11.50	6.3	1.9	3.3	3.0	0.1	2.5	0.0	-2.3	0.0	0	2.8	41
benzotrithiophene	21	1	1	P21/n	12.27	5.4	2.7	3.2	0.7	-0.8	4.8	0.0	-1.1	0.0	41	3.1	44
benzotrithiopheneA	21	2	1	P21	12.00	2.1	0.3	1.0	0.9	0.6	0.7	0.0	0.3	0.0	17	0.2	12
bithiophene	16	1	2	P21/c	11.52	2.8	1.5	1.9	2.7	0.6	0.1	0.0	1.0	0.0	0	1.4	31
BODIPY	21	1	1	C2/c	9.50	8.1	1.1	2.6	1.9	4.1	1.8	0.0	-0.4	0.0	27	1.1	24
BTBT	24	1	2	P21/c	10.75	2.3	0.4	1.0	0.9	0.3	1.1	0.0	0.2	0.0	0	0.9	7
C60	60	1	6	Pa-3	11.53	0.0	0.0	0.0	0.0	0.0	0.0	0.0	0.0	0.0	0	0.2	20
carbazole	22	1	2	Pnma	8.98	4.2	0.3	1.6	1.8	1.2	1.2	0.0	0.0	0.0	18	0.9	6
carbazoleNO2b	24	1	1	P21/n	9.57	2.5	1.4	1.0	0.6	2.4	-0.4	0.0	0.5	0.0	24	0.7	11
chrysene	30	1	2	C12/n1	9.18	3.4	0.5	1.3	0.8	1.4	1.2	0.0	0.3	0.0	0	0.6	6
coronene	36	1	2	P21/n	9.43	2.4	0.3	0.9	1.1	0.7	0.5	0.0	0.0	0.0	0	0.2	6
DATT	48	1	1	P21	9.82	4.5	1.1	1.5	1.1	2.7	0.7	0.0	-0.1	0.0	9	1.2	15
DBTTF	26	1	2	P21/c	11.59	2.5	0.3	1.2	0.9	0.8	0.9	0.0	0.2	0.0	0	0.3	11
dibenzoindigo	42	1	2	P21/n	9.34	0.6	1.1	1.5	-0.6	0.8	0.4	0.0	-0.2	0.0	0	0.5	30
DNBDT	42	1	2	P21/n	9.79	4.7	0.8	1.9	0.7	2.5	1.6	0.0	0.4	0.0	0	0.9	18
DNTT	36	1	1	P21	10.21	4.4	0.9	1.7	0.6	2.8	0.9	0.0	0.2	0.0	17	1.3	19
hexacene	42	2	2	P-1	8.88	4.5	1.4	2.4	-0.2	3.5	1.4	1.1	-0.0	-0.1	0	2.1	31
HMTTF	28	1	1	P21/c	9.99	6.6	1.5	3.1	1.7	3.8	1.0	0.0	0.5	0.0	5	0.7	18
ICZb	32	1	2	P21/c	8.93	3.6	1.2	2.1	1.9	0.3	1.6	0.0	1.2	0.0	0	1.5	10
ICZbCl2c	32	1	2	P21/c	10.06	-0.1	0.9	0.9	0.6	0.6	-1.1	0.0	0.4	0.0	0	0.4	13
IF12b	34	1	2	P21/n	8.96	5.8	1.1	2.2	1.5	2.5	1.6	0.0	-1.3	0.0	0	0.7	8
IF21a	34	1	1	P21/c	9.36	4.2	0.9	2.2	-0.3	3.1	1.4	0.0	0.2	0.0	4	0.6	13
indigo	30	1	2	P21/n	9.34	3.6	0.8	1.7	2.2	0.2	1.3	0.0	0.3	0.0	0	0.5	9
indigoCl2	30	1	2	P21/c	10.72	1.7	1.0	1.1	-0.3	1.4	1.0	0.0	0.6	0.0	0	0.8	7
naphthalene	18	1	2	P21/c	9.15	4.0	0.6	1.7	1.7	0.9	1.7	0.0	0.5	0.0	0	1.1	3
NDFPh	44	1	2	P21/c	9.56	7.3	1.3	2.6	1.6	4.4	1.3	0.0	0.4	0.0	0	1.6	18
NDI	26	2	2	P-1	9.68	3.3	0.8	1.3	1.1	1.3	0.6	0.7	0.6	-0.3	0	0.3	10
NDT	24	2	2	P21/c	10.75	7.2	2.2	4.6	4.5	0.8	1.7	0.0	-0.3	0.0	0	2.1	38
oligothiophene4	30	1	2	P21/a	11.55	5.5	2.4	3.2	0.7	1.6	2.5	0.0	-2.6	0.0	0	2.5	25
oligothiophene6	44	1	2	P21/a	11.53	4.9	1.9	3.4	1.5	1.2	2.5	0.0	1.9	0.0	0	1.7	26
OPV3CN	42	1	2	Pbca	9.88	4.7	0.6	2.2	1.0	2.4	1.2	0.0	0.0	0.0	0	1.0	38
PBBTZ	30	1	1	P21/n	10.44	1.9	0.7	0.6	1.0	0.1	0.8	0.0	-0.4	0.0	8	0.4	10
PDI	40	1	2	P21/n	9.35	3.4	0.6	1.4	0.9	0.9	1.6	0.0	0.8	0.0	0	0.4	25
pentacene	36	2	2	P-1	8.98	4.7	0.9	2.1	0.9	2.6	1.5	-0.9	-0.1	0.1	0	1.3	13
pentaceneN2Cl4	34	1	2	P21/n	11.61	2.4	0.5	0.7	1.5	0.4	0.4	0.0	-0.2	0.0	0	0.7	12
pentaceneN4O2	32	1	2	P21/n	9.62	5.6	1.4	1.7	3.8	0.7	1.0	0.0	-0.7	0.0	0	1.2	14
perylene	32	1	2	P21/c	8.98	4.6	0.8	1.4	1.3	2.6	0.6	0.0	-0.2	0.0	0	0.3	12
phenazine	22	1	2	P21/n	9.50	7.2	1.5	3.4	0.8	2.6	3.2	0.0	-1.2	0.0	0	1.4	10
PTCDA	38	1	2	P21/c	9.73	2.4	0.8	0.7	1.6	0.2	0.7	0.0	0.6	0.0	0	0.5	30
pyrene	26	1	1	P21/a	9.43	3.6	0.4	1.2	0.6	1.4	1.4	0.0	-0.1	0.0	16	0.4	7
quinacridone	36	1	2	P21/c	9.22	5.5	1.5	2.8	1.7	2.3	1.9	0.0	1.4	0.0	0	2.1	43
spirobidibenzosilole	41	1	2	P41212	10.25	3.6	0.3	1.3	1.3	1.3	0.9	0.0	0.0	0.0	32	0.8	22
spirobifluorene	41	1	1	P21/c	9.92	6.5	0.6	2.4	1.3	2.5	2.5	0.0	-0.4	0.0	14	0.4	22
stilbene	26	2	2	P21/a	9.16	4.3	0.2	1.6	1.2	1.7	1.4	0.0	-0.1	0.0	0	0.8	14
TCNQ	20	1	2	C2/c	12.08	3.7	1.3	1.2	0.9	2.8	0.0	0.0	0.4	0.0	0	0.6	12
TCNQ-F2	20	1	4	C2/m	12.26	4.2	1.0	1.4	0.6	3.0	0.6	0.0	0.2	0.0	0	0.0	12
TCNQ-F4	20	1	2	Pbca	13.13	4.2	0.3	1.6	1.8	0.8	1.5	0.0	0.0	0.0	0	0.6	13
tetracene	30	2	2	P-1	9.10	4.9	0.8	2.0	1.1	2.5	1.4	-0.7	0.3	0.0	0	1.3	13
thiazolothiazolePh	30	1	2	P21/c	10.49	6.1	0.8	3.0	2.1	1.7	1.8	0.0	-1.1	0.0	0	1.1	21
TPP	34	1	1	P21/c	9.89	3.8	0.7	1.5	0.8	0.9	2.1	0.0	0.6	0.0	13	0.6	14
triazine	9	1	2	C2/c	9.80	4.2	1.9	2.7	4.1	1.0	1.2	0.0	1.8	0.0	9	1.9	3
triphenylbenzene	42	1	1	Pna21	9.33	4.5	0.9	1.3	2.8	0.7	0.9	0.0	0.0	0.0	7	0.5	38
triphenylene	30	1	1	P212121	9.06	4.7	0.2	1.9	1.4	1.8	1.4	0.0	0.0	0.0	9	0.3	10
triphenyltriazine	39	1	1	P21/c	9.18	4.5	0.8	2.1	1.2	2.2	1.1	0.0	0.0	0.0	9	0.3	13
triptycene	34	1	1	P212121	9.57	4.3	0.0	1.7	1.4	1.5	1.4	0.0	0.0	0.0	12	0.2	15
TTAa	36	1	2	P21/n	11.13	1.3	0.6	1.0	0.7	-0.3	0.9	0.0	-0.3	0.0	0	0.5	13
TTAb	36	1	2	P21/n	11.14	2.9	0.9	1.6	0.5	0.5	1.9	0.0	0.1	0.0	0	0.4	16
TTF	14	1	2	P21/c	13.52	6.3	0.9	2.3	1.4	3.4	1.7	0.0	0.7	0.0	0	0.7	9
TTPa	34	1	2	P21/n	11.51	2.1	0.4	1.0	0.9	0.3	0.9	0.0	-0.4	0.0	0	0.4	11
TTPb	34	2	2	P21/a	11.67	1.9	0.6	0.6	0.7	1.1	0.2	0.0	0.1	0.0	0	1.1	20
TTPc	34	1	1	P21	11.76	3.5	1.7	2.6	0.7	-0.4	3.0	0.0	-0.5	0.0	8	1.2	18
TTTT	18	1	2	P21/n	12.57	3.4	0.7	1.2	0.3	1.8	1.3	0.0	0.2	0.0	0	0.6	7

Table S12: Comparison experiment wrt PBE-D3paw900 [URL].

system	na1	no	ns	SG	V1	dV1	dSh	dTv	da	db	dc	dalp	dbet	dgam	dr	phi	dev
str	#	#	#	str	Ao3	%	%	%	%	%	%	deg	deg	deg	mAo	deg	mAo
median						1.8	0.7	1.0	0.5	0.7	0.5	0.0	0.0	0.0	0	0.6	18
mean						1.6	0.8	1.2	0.5	0.7	0.4	-0.0	0.0	0.0	4	0.8	20
standard dev						2.0	0.6	0.8	0.9	1.0	1.0	0.1	0.5	0.1	7	0.6	11
lower decile						-1.2	0.2	0.5	-0.5	-0.5	-0.9	0.0	-0.5	0.0	0	0.2	9
upper decile						4.0	1.5	2.2	1.4	1.9	1.6	0.0	0.7	0.0	13	1.7	37
min						-5.8	0.0	0.2	-2.2	-3.0	-3.3	-0.3	-1.9	-0.5	0	0.1	3
max						6.4	3.1	4.6	4.7	3.5	2.9	0.4	1.3	0.3	37	2.6	52
anthracene	24	1	2	P21/c	9.38	1.4	0.3	0.5	0.3	1.0	0.2	0.0	0.1	0.0	0	0.4	8
anthraquinone	24	1	2	P21/c	9.67	1.8	0.9	0.5	0.6	1.4	-0.2	0.0	0.0	0.0	0	0.7	12
benzene	12	1	2	Pbca	9.54	3.7	0.1	1.3	1.2	1.4	1.1	0.0	0.0	0.0	0	0.5	3
benzeneF6	12	2	1	P21/n	13.26	-5.8	0.7	2.5	-1.1	-3.0	-1.8	0.0	-0.1	0.0	0	1.0	21
benzeneO2	12	1	2	P21/c	10.70	2.4	1.3	1.7	-0.6	2.2	1.1	0.0	1.0	0.0	0	2.2	14
benzodithiopheneO2	18	1	2	P21/n	11.82	3.4	0.5	1.2	1.9	0.6	0.9	0.0	-0.3	0.0	0	0.8	40
benzotrithiophene	21	1	1	P21/n	12.45	4.0	1.4	1.9	0.5	0.2	2.9	0.0	-0.4	0.0	12	1.7	48
benzotrithiopheneA	21	2	1	P21	12.11	1.1	0.2	0.5	0.6	0.2	0.4	0.0	0.1	0.0	37	0.4	13
bithiophene	16	1	2	P21/c	11.72	1.1	1.5	1.5	1.7	0.9	-1.3	0.0	0.5	0.0	0	1.8	32
BODIPY	21	1	1	C2/c	9.97	3.0	0.8	1.5	-0.1	1.3	1.6	0.0	-0.4	0.0	8	1.0	25
BTBT	24	1	2	P21/c	10.91	0.7	0.6	0.7	0.1	-0.4	1.1	0.0	0.2	0.0	0	1.1	8
C60	60	1	6	Pa-3	11.70	-1.4	0.0	0.5	-0.5	-0.5	-0.5	0.0	0.0	0.0	0	0.2	18
carbazole	22	1	2	Pnma	9.21	1.6	0.1	0.6	0.6	0.4	0.6	0.0	0.0	0.0	8	0.2	10
carbazoleNO2b	24	1	1	P21/n	9.96	-1.5	1.3	1.6	-0.8	0.9	-1.2	0.0	1.0	0.0	30	1.2	19
chrysene	30	1	2	C12/n1	9.38	1.1	0.8	0.6	-0.1	1.3	0.1	0.0	0.5	0.0	0	0.3	11
coronene	36	1	2	P21/n	9.57	0.9	0.6	0.9	0.5	-0.4	0.8	0.0	-0.0	0.0	0	0.1	10
DATT	48	1	1	P21	9.98	2.8	0.9	0.9	1.0	1.5	0.2	0.0	-0.5	0.0	15	0.7	24
DBTTF	26	1	2	P21/c	11.69	1.7	0.4	0.9	0.7	0.6	0.6	0.0	0.3	0.0	0	0.1	13
dibenzoindigo	42	1	2	P21/n	9.59	-2.0	3.1	2.1	-2.2	1.1	-0.9	0.0	0.7	0.0	0	1.7	52
DNBDT	42	1	2	P21/n	9.94	3.1	0.6	1.3	0.5	1.7	1.0	0.0	0.3	0.0	0	0.7	11
DNTT	36	1	1	P21	10.39	2.6	0.7	0.9	0.8	1.5	0.3	0.0	0.4	0.0	9	0.7	30
hexacene	42	2	2	P-1	9.05	2.5	1.2	1.8	-0.8	2.1	1.2	0.4	-1.0	-0.5	0	1.7	25
HMTTF	28	1	1	P21/c	10.18	4.7	1.8	2.8	1.1	3.5	0.1	0.0	0.3	0.0	12	0.2	18
ICZb	32	1	2	P21/c	9.16	1.0	0.2	0.4	0.3	0.5	0.3	0.0	0.3	0.0	0	0.4	11
ICZbCl2c	32	1	2	P21/c	10.34	-2.8	1.6	1.7	-0.1	0.6	-3.3	0.0	-0.2	0.0	0	1.7	24
IF12b	34	1	2	P21/n	9.17	3.4	1.0	1.8	0.5	1.3	1.5	0.0	-1.0	0.0	0	0.8	16
IF21a	34	1	1	P21/c	9.58	1.8	0.6	0.6	0.7	0.9	0.2	0.0	0.3	0.0	10	0.6	10
indigo	30	1	2	P21/n	9.59	0.9	0.4	0.6	0.6	-0.2	0.6	0.0	0.0	0.0	0	0.2	22
indigoCl2	30	1	2	P21/c	11.01	-0.9	1.3	1.5	-1.6	1.0	-0.5	0.0	-0.3	0.0	0	1.7	20
naphthalene	18	1	2	P21/c	9.41	1.1	0.2	0.4	0.2	0.6	0.4	0.0	0.1	0.0	0	0.4	7
NDFPh	44	1	2	P21/c	9.86	4.0	0.9	1.4	0.7	2.2	1.2	0.0	0.5	0.0	0	1.0	23
NDI	26	2	2	P-1	10.07	-0.7	1.2	1.3	1.4	-0.6	-1.4	-0.2	-0.3	0.2	0	1.3	13
NDT	24	2	2	P21/c	10.92	5.6	2.9	4.6	4.7	-0.0	0.8	0.0	-0.6	0.0	0	2.6	39
oligothiophene4	30	1	2	P21/a	11.73	3.8	1.9	2.3	-0.2	1.8	1.7	0.0	-1.9	0.0	0	1.8	31
oligothiophene6	44	1	2	P21/a	11.70	3.3	1.2	2.2	0.6	1.4	1.5	0.0	1.3	0.0	0	0.9	32
OPV3CN	42	1	2	Pbca	10.12	2.2	1.0	0.6	0.7	1.4	0.1	0.0	0.0	0.0	0	0.4	50
PBBTZ	30	1	1	P21/n	10.57	0.7	0.6	0.5	-0.1	-0.1	0.8	0.0	-0.3	0.0	13	0.5	21
PDI	40	1	2	P21/n	9.64	0.2	0.8	0.8	0.8	-0.8	0.2	0.0	0.2	0.0	0	1.0	27
pentacene	36	2	2	P-1	9.17	2.6	0.7	1.4	0.4	1.2	1.1	-0.3	-0.8	0.3	0	0.9	9
pentaceneN2Cl4	34	1	2	P21/n	11.88	0.1	0.8	0.8	-0.1	0.7	-0.5	0.0	-0.1	0.0	0	0.5	11
pentaceneN4O2	32	1	2	P21/n	9.92	2.4	1.0	0.7	2.2	0.1	0.2	0.0	0.7	0.0	0	1.5	17
perylene	32	1	2	P21/c	9.16	2.5	1.8	1.6	0.2	3.0	-0.9	0.0	-0.9	0.0	0	0.8	17
phenazine	22	1	2	P21/n	9.75	4.5	0.5	2.0	1.7	0.8	1.8	0.0	-0.2	0.0	0	2.0	18
PTCDA	38	1	2	P21/c	10.10	-1.3	0.6	1.1	0.1	-0.9	-0.5	0.0	-0.4	0.0	0	0.4	37
pyrene	26	1	1	P21/a	9.60	1.7	0.9	1.0	-0.4	1.5	0.6	0.0	-0.3	0.0	11	0.9	12
quinacridone	36	1	2	P21/c	9.47	2.7	1.3	1.8	1.1	0.9	1.0	0.0	1.1	0.0	0	1.0	45
spirobidibenzosilole	41	1	2	P41212	10.43	1.8	0.2	0.9	0.5	0.5	0.8	0.0	0.0	0.0	5	0.4	18
spirobifluorene	41	1	1	P21/c	10.11	4.4	0.4	1.7	0.8	1.9	1.6	0.0	-0.2	0.0	15	0.4	23
stilbene	26	2	2	P21/a	9.37	2.0	0.4	0.9	1.1	0.7	0.4	0.0	0.2	0.0	0	0.5	10
TCNQ	20	1	2	C2/c	12.42	0.8	1.0	0.9	0.4	1.1	-0.5	0.0	0.8	0.0	0	1.3	17
TCNQ-F2	20	1	4	C2/m	12.84	-0.5	0.5	0.5	-0.0	-0.5	0.2	0.0	0.5	0.0	0	0.2	17
TCNQ-F4	20	1	2	Pbca	13.90	-1.7	1.2	1.1	-1.2	-1.3	0.8	0.0	0.0	0.0	0	0.8	21
tetracene	30	2	2	P-1	9.30	2.7	0.3	1.1	1.0	1.0	0.9	-0.1	-0.3	0.2	0	0.3	11
thiazolothiazolePh	30	1	2	P21/c	10.70	4.0	0.5	2.4	1.1	1.1	1.5	0.0	-0.4	0.0	0	1.1	21
TPP	34	1	1	P21/c	10.07	2.0	1.0	1.1	0.5	-0.2	1.7	0.0	0.6	0.0	21	0.1	26
triazine	9	1	2	C2/c	10.20	0.0	0.5	0.5	0.4	0.1	-0.7	0.0	-0.2	0.0	4	0.4	5
triphenylbenzene	42	1	1	Pna21	9.55	2.2	0.6	0.7	1.6	0.3	0.3	0.0	0.0	0.0	3	0.3	25
triphenylene	30	1	1	P212121	9.25	2.5	0.4	1.2	0.7	0.6	1.2	0.0	0.0	0.0	15	0.3	11
triphenyltriazine	39	1	1	P21/c	9.43	1.8	0.2	1.0	0.6	0.6	0.5	0.0	-0.1	0.0	11	0.1	12
triptycene	34	1	1	P212121	9.77	2.2	0.2	0.7	0.7	1.0	0.5	0.0	0.0	0.0	7	0.1	4
TTAa	36	1	2	P21/n	11.27	0.1	0.2	0.2	-0.0	-0.0	0.1	0.0	0.2	0.0	0	0.3	19
TTAb	36	1	2	P21/n	11.25	1.9	0.4	0.8	0.2	0.7	0.9	0.0	-0.2	0.0	0	0.2	10
TTF	14	1	2	P21/c	13.51	6.4	0.7	2.8	1.3	2.9	2.3	0.0	0.5	0.0	0	1.2	14
TTPa	34	1	2	P21/n	11.67	0.6	0.5	0.5	0.2	0.4	0.1	0.0	0.5	0.0	0	0.4	16
TTPb	34	2	2	P21/a	11.83	0.6	0.5	0.6	0.6	0.2	-0.1	0.0	0.2	0.0	0	0.6	29
TTPc	34	1	1	P21	11.92	2.0	0.9	1.4	0.3	0.1	1.6	0.0	-0.2	0.0	8	0.4	18
TTTT	18	1	2	P21/n	12.70	2.3	0.7	0.7	0.0	1.6	0.7	0.0	0.0	0.0	0	0.6	16

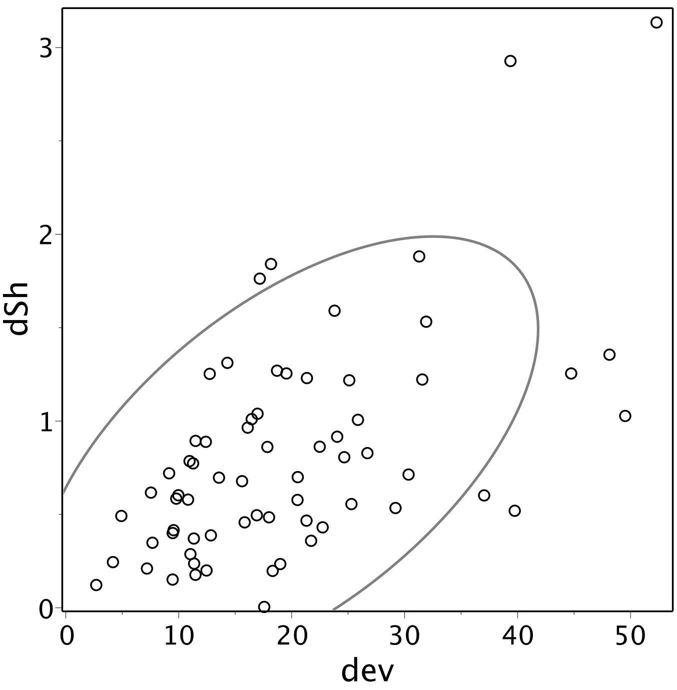

Figure S3: Comparison experiment wrt PBE-D3paw900: dev-dSh correlation [URL].

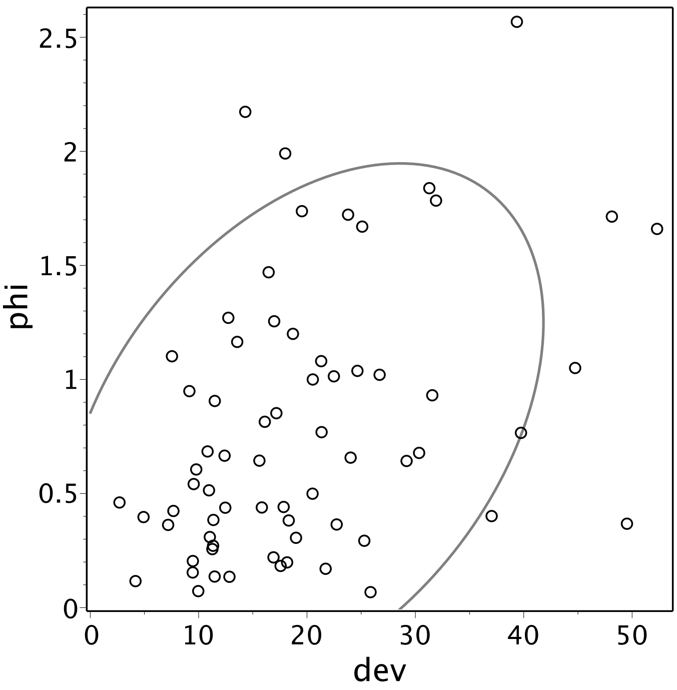

Figure S4: Comparison experiment wrt PBE-D3paw900: dev-phi correlation [URL].

Table S13: Comparison R2SCAN wrt PBE-D3 [URL].

system	na1	no	ns	SG	V1	dV1	dSh	dTv	da	db	dc	dalp	dbet	dgam	dr	phi	dev
str	#	#	#	str	Ao3	%	%	%	%	%	%	deg	deg	deg	mAo	deg	mAo
median						-2.2	0.7	1.1	-0.7	-0.7	-0.9	0.0	-0.0	0.0	0	0.8	16
mean						-2.4	0.8	1.2	-0.8	-0.8	-0.9	-0.0	-0.0	0.0	5	0.9	16
standard dev						1.4	0.5	0.6	0.8	1.1	0.7	0.2	0.6	0.1	10	0.6	5
lower decile						-3.9	0.3	0.6	-1.6	-1.8	-1.8	0.0	-0.8	0.0	0	0.2	10
upper decile						-1.3	1.4	1.9	-0.0	0.3	-0.2	0.0	0.7	0.0	20	1.6	23
min						-10.1	0.0	0.4	-3.5	-6.2	-4.0	-1.0	-1.9	-0.3	0	0.0	5
max						0.1	2.3	4.3	1.0	1.1	0.6	0.6	2.1	0.5	52	2.5	28
anthracene	24	1	2	P21/c	9.38	-2.4	0.7	1.2	-1.1	0.1	-1.6	0.0	-0.2	0.0	0	1.3	10
anthraquinone	24	1	2	P21/c	9.67	-3.1	0.6	1.8	-0.5	-1.2	-1.5	0.0	-0.1	0.0	0	0.9	12
benzene	12	1	2	Pbca	9.54	-3.1	0.7	1.3	0.0	-1.4	-1.8	0.0	0.0	0.0	0	1.5	5
benzeneF6	12	2	1	P21/n	13.26	-10.1	1.9	4.3	-1.6	-6.2	-2.6	0.0	-0.2	0.0	0	2.5	13
benzeneO2	12	1	2	P21/c	10.70	-4.9	2.3	2.9	-2.2	1.1	-4.0	0.0	-0.9	0.0	0	2.1	10
benzodithiopheneO2	18	1	2	P21/n	11.82	-2.7	1.8	2.3	-1.0	0.5	-1.6	0.0	2.1	0.0	0	2.3	11
benzotrithiophene	21	1	1	P21/n	12.45	-1.4	1.4	1.4	-0.2	1.0	-1.8	0.0	0.6	0.0	30	1.4	12
benzotrithiopheneA	21	2	1	P21	12.11	-0.9	0.2	0.4	-0.3	-0.4	-0.4	0.0	-0.2	0.0	20	0.6	12
bithiophene	16	1	2	P21/c	11.72	-1.7	0.8	1.2	-1.0	0.4	-1.3	0.0	-0.5	0.0	0	1.5	9
BODIPY	21	1	1	C2/c	9.97	-4.7	1.3	1.6	-1.9	-2.7	-0.2	0.0	-0.0	0.0	28	1.6	12
BTBT	24	1	2	P21/c	10.91	-1.5	0.4	0.8	-0.8	-0.7	0.0	0.0	-0.0	0.0	0	0.3	12
C60	60	1	6	Pa-3	11.70	-1.4	0.0	0.5	-0.5	-0.5	-0.5	0.0	0.0	0.0	0	0.0	15
carbazole	22	1	2	Pnma	9.21	-2.5	0.2	1.0	-1.2	-0.7	-0.6	0.0	0.0	0.0	19	1.1	11
carbazoleNO2b	24	1	1	P21/n	9.96	-3.9	0.6	1.4	-1.4	-1.5	-0.9	0.0	0.5	0.0	52	0.8	13
chrysene	30	1	2	C12/n1	9.38	-2.2	0.4	1.3	-0.9	-0.1	-1.1	0.0	0.1	0.0	0	0.3	11
coronene	36	1	2	P21/n	9.57	-1.5	0.8	0.6	-0.6	-1.1	0.3	0.0	-0.1	0.0	0	0.3	12
DATT	48	1	1	P21	9.98	-1.7	0.4	0.8	-0.0	-1.2	-0.4	0.0	-0.4	0.0	7	0.7	23
DBTTF	26	1	2	P21/c	11.69	-0.8	0.1	0.4	-0.2	-0.2	-0.3	0.0	0.1	0.0	0	0.2	15
dibenzoindigo	42	1	2	P21/n	9.59	-2.6	2.1	0.9	-1.6	0.3	-1.3	0.0	0.8	0.0	0	1.3	24
DNBDT	42	1	2	P21/n	9.94	-1.5	0.3	0.7	-0.2	-0.8	-0.6	0.0	-0.1	0.0	0	0.3	19
DNTT	36	1	1	P21	10.39	-1.7	0.4	0.9	0.2	-1.3	-0.6	0.0	0.2	0.0	10	0.7	18
hexacene	42	2	2	P-1	9.05	-2.0	1.0	1.2	-0.6	-1.4	-0.3	-0.7	-1.0	-0.3	0	1.6	18
HMTTF	28	1	1	P21/c	10.18	-1.9	0.3	0.7	-0.7	-0.3	-0.9	0.0	-0.2	0.0	16	0.5	15
ICZb	32	1	2	P21/c	9.16	-2.5	1.1	1.8	-1.6	0.2	-1.3	0.0	-0.9	0.0	0	1.8	16
ICZbCl2c	32	1	2	P21/c	10.34	-2.7	0.9	1.4	-0.7	0.0	-2.2	0.0	-0.6	0.0	0	1.7	17
IF12b	34	1	2	P21/n	9.17	-2.2	0.7	0.7	-1.0	-1.1	-0.1	0.0	0.3	0.0	0	0.1	15
IF21a	34	1	1	P21/c	9.58	-2.3	1.1	1.8	1.0	-2.1	-1.2	0.0	0.1	0.0	12	1.1	19
indigo	30	1	2	P21/n	9.59	-2.6	0.6	1.2	-1.6	-0.4	-0.7	0.0	-0.3	0.0	0	0.4	19
indigoCl2	30	1	2	P21/c	11.01	-2.6	1.1	1.8	-1.3	-0.4	-1.4	0.0	-0.9	0.0	0	1.6	22
naphthalene	18	1	2	P21/c	9.41	-2.8	0.6	1.3	-1.6	-0.3	-1.2	0.0	-0.3	0.0	0	1.0	8
NDFPh	44	1	2	P21/c	9.86	-3.0	0.6	1.3	-0.9	-2.0	-0.1	0.0	0.1	0.0	0	0.7	24
NDI	26	2	2	P-1	10.07	-3.8	1.5	2.0	0.3	-1.8	-2.1	-1.0	-1.0	0.5	0	1.5	18
NDT	24	2	2	P21/c	10.92	-1.5	0.7	0.6	0.1	-0.8	-0.9	0.0	-0.2	0.0	0	0.6	11
oligothiophene4	30	1	2	P21/a	11.73	-1.6	0.7	1.0	-0.8	0.2	-0.8	0.0	0.7	0.0	0	1.0	17
oligothiophene6	44	1	2	P21/a	11.70	-1.5	0.8	1.3	-0.9	0.2	-0.9	0.0	-0.6	0.0	0	1.1	25
OPV3CN	42	1	2	Pbca	10.12	-2.4	0.5	1.9	-0.4	-1.0	-1.1	0.0	0.0	0.0	0	0.7	26
PBBTZ	30	1	1	P21/n	10.57	-1.3	0.4	0.4	-1.0	-0.2	0.0	0.0	0.1	0.0	12	0.2	16
PDI	40	1	2	P21/n	9.64	-3.1	0.7	1.8	-0.1	-1.6	-1.4	0.0	-0.5	0.0	0	1.1	23
pentacene	36	2	2	P-1	9.17	-2.0	0.8	1.1	-0.5	-1.4	-0.4	0.5	-0.7	0.2	0	1.4	16
pentaceneN2Cl4	34	1	2	P21/n	11.88	-2.3	0.8	1.1	-1.6	0.3	-1.0	0.0	0.1	0.0	0	0.7	16
pentaceneN4O2	32	1	2	P21/n	9.92	-3.0	0.7	1.3	-1.6	-0.6	-0.9	0.0	1.4	0.0	0	1.1	19
perylene	32	1	2	P21/c	9.16	-2.0	1.0	1.5	-1.1	0.4	-1.5	0.0	-0.6	0.0	0	0.6	10
phenazine	22	1	2	P21/n	9.75	-2.6	1.4	1.7	0.9	-1.7	-1.4	0.0	1.0	0.0	0	1.1	10
PTCDA	38	1	2	P21/c	10.10	-3.6	0.4	1.6	-1.5	-1.1	-1.2	0.0	-0.9	0.0	0	0.4	22
pyrene	26	1	1	P21/a	9.60	-1.8	0.5	0.9	-1.0	0.1	-0.8	0.0	-0.2	0.0	20	0.8	10
quinacridone	36	1	2	P21/c	9.47	-2.7	0.4	1.0	-0.6	-1.3	-0.9	0.0	-0.3	0.0	0	1.2	17
spirobidibenzosilole	41	1	2	P41212	10.43	-1.8	0.5	0.5	-0.8	-0.8	-0.2	0.0	0.0	0.0	27	0.4	25
spirobifluorene	41	1	1	P21/c	10.11	-1.9	0.3	0.8	-0.5	-0.5	-0.9	0.0	0.2	0.0	6	0.3	13
stilbene	26	2	2	P21/a	9.37	-2.2	0.6	1.0	-0.1	-1.0	-1.0	0.0	0.3	0.0	0	0.4	14
TCNQ	20	1	2	C2/c	12.42	-2.7	0.6	0.9	-0.5	-1.6	-0.6	0.0	0.4	0.0	0	0.7	18
TCNQ-F2	20	1	4	C2/m	12.84	-4.5	1.3	1.6	-0.6	-3.4	-0.4	0.0	0.3	0.0	0	0.2	18
TCNQ-F4	20	1	2	Pbca	13.90	-5.6	1.2	1.9	-2.9	-2.1	-0.7	0.0	0.0	0.0	0	0.9	19
tetracene	30	2	2	P-1	9.30	-2.1	0.8	1.1	-0.1	-1.4	-0.5	0.6	-0.5	0.1	0	1.2	13
thiazolothiazolePh	30	1	2	P21/c	10.70	-2.0	0.7	0.7	-1.0	-0.5	-0.3	0.0	0.7	0.0	0	1.0	18
TPP	34	1	1	P21/c	10.07	-1.8	0.5	0.9	-0.3	-1.2	-0.3	0.0	-0.0	0.0	23	0.7	28
triazine	9	1	2	C2/c	10.20	-4.0	1.8	2.6	-3.5	-0.9	-1.9	0.0	-1.9	0.0	5	1.5	6
triphenylbenzene	42	1	1	Pna21	9.55	-2.2	0.4	0.7	-1.2	-0.4	-0.6	0.0	0.0	0.0	6	0.2	22
triphenylene	30	1	1	P212121	9.25	-2.1	0.6	0.9	-0.7	-1.2	-0.2	0.0	0.0	0.0	13	0.3	11
triphenyltriazine	39	1	1	P21/c	9.43	-2.6	0.6	1.1	-0.6	-1.5	-0.6	0.0	-0.1	0.0	9	0.2	17
triptycene	34	1	1	P212121	9.77	-2.0	0.2	1.0	-0.7	-0.5	-0.9	0.0	0.0	0.0	8	0.1	12
TTAa	36	1	2	P21/n	11.27	-1.2	0.7	1.0	-0.7	0.3	-0.8	0.0	0.6	0.0	0	0.7	17
TTAb	36	1	2	P21/n	11.25	-1.0	0.6	0.8	-0.2	0.2	-1.0	0.0	-0.2	0.0	0	0.4	16
TTF	14	1	2	P21/c	13.51	0.1	0.6	0.6	-0.0	-0.5	0.6	0.0	-0.2	0.0	0	0.9	8
TTPa	34	1	2	P21/n	11.67	-1.4	0.9	1.1	-0.7	0.1	-0.7	0.0	0.8	0.0	0	0.8	16
TTPb	34	2	2	P21/a	11.83	-1.3	0.4	0.4	-0.1	-0.9	-0.3	0.0	0.2	0.0	0	0.6	17
TTPc	34	1	1	P21	11.92	-1.4	0.9	1.2	-0.4	0.5	-1.4	0.0	0.3	0.0	6	0.8	18
TTTT	18	1	2	P21/n	12.70	-1.0	0.3	0.6	-0.3	-0.2	-0.6	0.0	-0.2	0.0	0	0.2	11

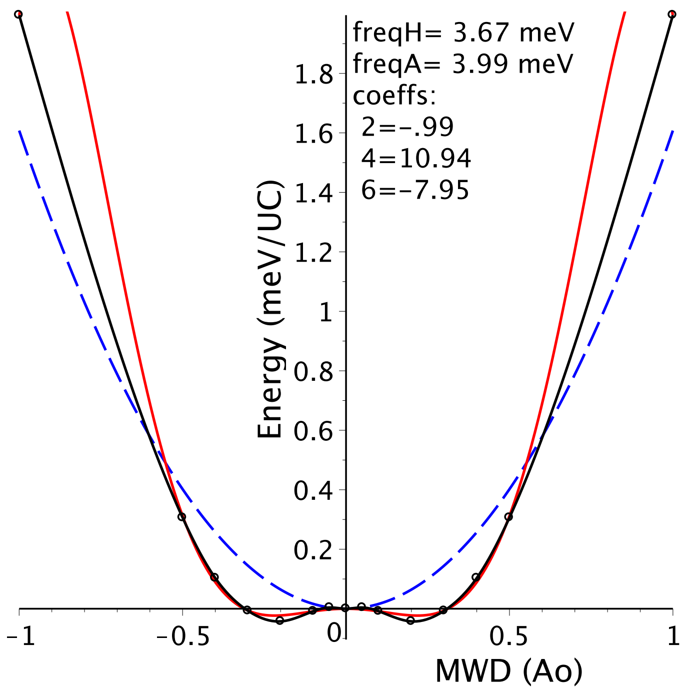

Figure S5: PES scans of imaginary frequencies: benzeneO2 by PBE-D3 [URL].

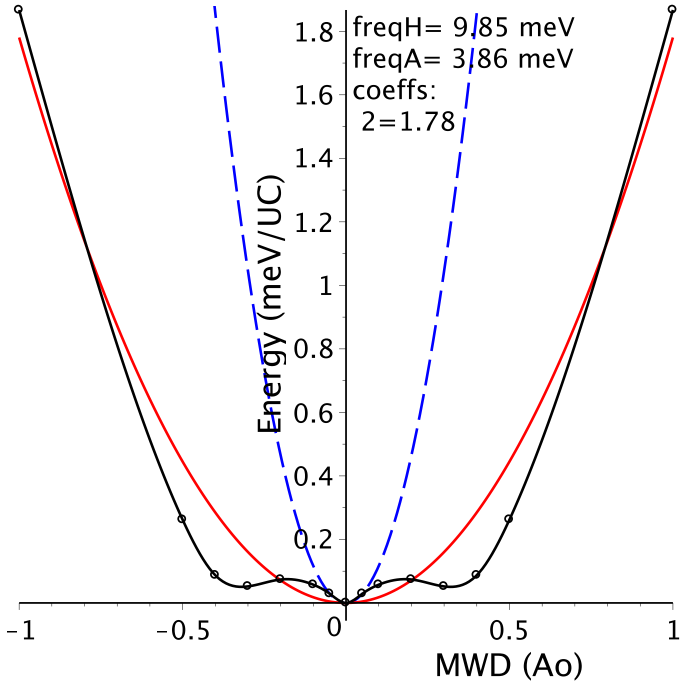

Figure S6: PES scans of imaginary frequencies: carbazoleNO2b by PBE-D3 [URL].

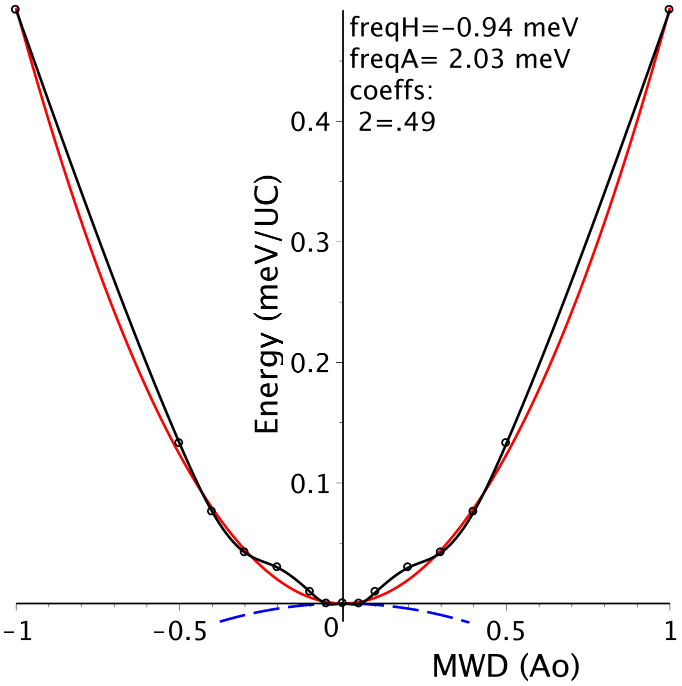

Figure S7: PES scans of imaginary frequencies: IF21a by PBE-D3 [URL].

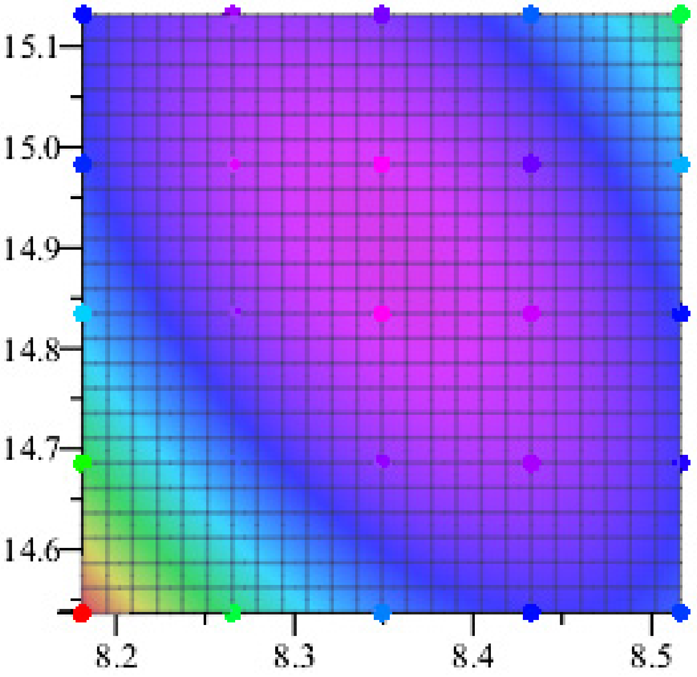

Figure S8: PES scans of unit cell parameters: TPP 2D scan in ab-plane plane by PBE-D3 [URL].

Table S14: PES scans of unit cell parameters: TPP 2D scan in ab-plane plane by PBE-D3 [URL].

2D cg2-scan of cryst_PBE-D3paw900 (opttol=1e-3):

x1	x2	E	V1	a	b	c	alp	bet	gam	dE	res(ueV)	file
8.1820	14.5380	-6.60770	9.6748	8.182	14.538	11.063	90.000	90.919	90.000	757	-7	11
8.1820	14.6863	-6.60799	9.7735	8.182	14.686	11.063	90.000	90.919	90.000	471	-1	12
8.1820	14.8346	-6.60817	9.8722	8.182	14.835	11.063	90.000	90.919	90.000	284	-6	13
8.1820	14.9830	-6.60827	9.9710	8.182	14.983	11.063	90.000	90.919	90.000	185	-9	14
8.1820	15.1313	-6.60829	10.0697	8.182	15.131	11.063	90.000	90.919	90.000	168	-3	15
8.2655	14.5380	-6.60803	9.7735	8.265	14.538	11.063	90.000	90.928	90.000	425	8	21
8.2655	14.6863	-6.60825	9.8733	8.265	14.686	11.063	90.000	90.928	90.000	204	15	22
8.2655	14.8346	-6.60838	9.9730	8.265	14.835	11.063	90.000	90.928	90.000	79	10	23
8.2655	14.9830	-6.60842	10.0727	8.265	14.983	11.063	90.000	90.928	90.000	34	8	24
8.2655	15.1313	-6.60839	10.1724	8.265	15.131	11.063	90.000	90.928	90.000	68	11	25
8.3490	14.5380	-6.60822	9.8722	8.349	14.538	11.063	90.000	90.938	90.000	237	-2	31
8.3490	14.6863	-6.60838	9.9730	8.349	14.686	11.063	90.000	90.938	90.000	79	4	32
8.3490	14.8346	-6.60845	10.0737	8.349	14.835	11.063	90.000	90.938	90.000	9	-1	33
8.3490	14.9830	-6.60844	10.1745	8.349	14.983	11.063	90.000	90.938	90.000	16	-4	34
8.3490	15.1313	-6.60836	10.2752	8.349	15.131	11.063	90.000	90.938	90.000	94	1	35
8.4324	14.5380	-6.60829	9.9710	8.432	14.538	11.063	90.000	90.947	90.000	170	-12	41
8.4324	14.6863	-6.60839	10.0727	8.432	14.686	11.063	90.000	90.947	90.000	67	-6	42
8.4324	14.8346	-6.60841	10.1745	8.432	14.835	11.063	90.000	90.947	90.000	47	-10	43
8.4324	14.9830	-6.60836	10.2762	8.432	14.983	11.063	90.000	90.947	90.000	100	-13	44
8.4324	15.1313	-6.60824	10.3779	8.432	15.131	11.063	90.000	90.947	90.000	219	-7	45
8.5159	14.5380	-6.60826	10.0697	8.516	14.538	11.063	90.000	90.957	90.000	198	3	51
8.5159	14.6863	-6.60831	10.1724	8.516	14.686	11.063	90.000	90.957	90.000	143	9	52
8.5159	14.8346	-6.60829	10.2752	8.516	14.835	11.063	90.000	90.957	90.000	170	5	53
8.5159	14.9830	-6.60819	10.3779	8.516	14.983	11.063	90.000	90.957	90.000	266	1	54
8.5159	15.1313	-6.60803	10.4807	8.516	15.131	11.063	90.000	90.957	90.000	423	6	55
8.3530	14.8963	-6.60846	10.1205	8.353	14.896	11.063	90.000	90.938	90.000			predicted
8.3490	14.8346	-6.60845	10.0737	8.349	14.835	11.063	90.000	90.938	90.000	9	-1	cg3

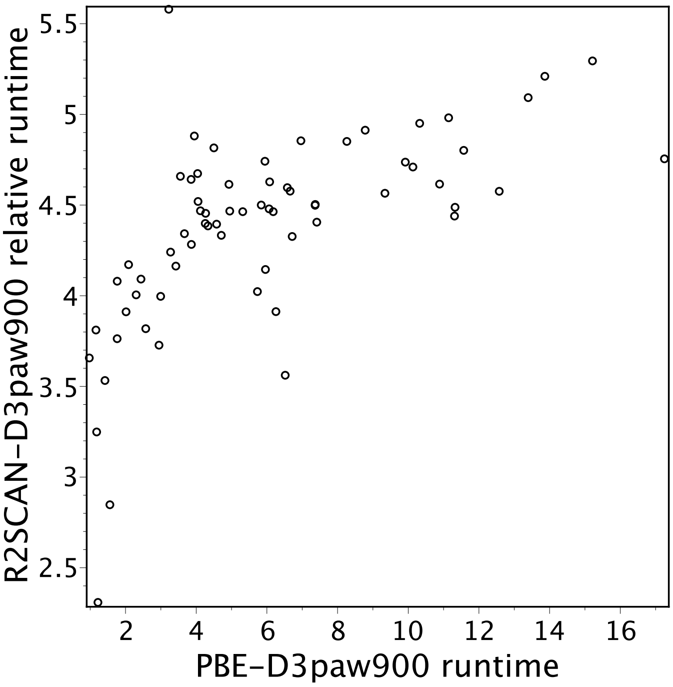

Figure S9: Runtime: R2SCAN vs PBE [URL].

Table S15: Comparison medium accuracy wrt high accuracy PBE-D3paw900 [URL].

system	na1	no	ns	SG	V1	dV1	dSh	dTv	da	db	dc	dalp	dbet	dgam	dr	phi	dev
str	#	#	#	str	Ao3	%	%	%	%	%	%	deg	deg	deg	mAo	deg	mAo
median						0.4	0.6	0.6	0.1	0.3	0.2	0.0	-0.0	0.0	0	0.5	3
mean						0.4	0.7	0.8	0.1	0.2	0.1	-0.0	-0.1	-0.0	3	0.6	5
standard dev						0.4	0.6	0.6	0.7	0.8	0.6	0.2	0.4	0.0	7	0.5	5
lower decile						-0.2	0.2	0.3	-0.9	-0.7	-0.7	0.0	-0.6	0.0	0	0.2	1
upper decile						0.9	1.3	1.4	0.7	1.2	0.7	0.0	0.5	0.0	12	1.3	10
min						-0.8	0.0	0.1	-1.5	-2.4	-2.0	-1.0	-0.9	-0.3	0	0.1	0
max						1.4	3.2	3.2	3.0	2.9	1.2	0.7	0.9	0.2	32	2.6	29
anthracene	24	1	2	P21/c	9.38	1.2	1.2	1.1	-0.2	1.8	-0.7	0.0	-0.7	0.0	0	0.7	1
anthraquinone	24	1	2	P21/c	9.67	-0.1	0.5	0.5	0.6	-0.9	-0.0	0.0	-0.3	0.0	0	0.4	2
benzene	12	1	2	Pbca	9.54	1.4	0.2	0.5	0.2	0.6	0.6	0.0	0.0	0.0	0	0.5	0
benzeneF6	12	2	1	P21/n	13.26	-0.7	0.4	0.5	0.3	-0.8	-0.2	0.0	-0.0	0.0	0	0.6	1
benzeneO2	12	1	2	P21/c	10.70	0.4	0.9	0.9	-0.3	1.0	-0.1	0.0	0.9	0.0	0	1.8	1
benzodithiopheneO2	18	1	2	P21/n	11.82	0.6	0.5	0.6	-0.5	0.6	0.5	0.0	-0.2	0.0	0	0.4	3
benzotrithiophene	21	1	1	P21/n	12.45	0.1	0.5	0.5	-0.3	-0.2	0.7	0.0	0.2	0.0	9	0.6	6
benzotrithiopheneA	21	2	1	P21	12.11	0.3	0.6	0.6	0.2	0.4	-0.4	0.0	-0.4	0.0	32	0.4	4
bithiophene	16	1	2	P21/c	11.72	0.4	1.6	1.5	0.9	0.9	-1.7	0.0	-0.7	0.0	0	2.2	1
BODIPY	21	1	1	C2/c	9.97	-0.2	0.9	0.9	-0.8	-0.4	0.9	0.0	-0.4	0.0	23	1.1	18
BTBT	24	1	2	P21/c	10.91	0.6	0.2	0.4	0.3	0.0	0.3	0.0	0.1	0.0	0	0.2	1
C60	60	1	6	Pa-3	11.70	-0.3	0.0	0.1	-0.1	-0.1	-0.1	0.0	0.0	0.0	0	0.2	0
carbazole	22	1	2	Pnma	9.21	0.3	0.7	0.8	-0.9	0.5	0.6	0.0	0.0	0.0	6	0.7	1
carbazoleNO2b	24	1	1	P21/n	9.96	-0.2	0.9	1.0	-0.4	1.0	-0.5	0.0	0.8	0.0	2	0.8	7
chrysene	30	1	2	C12/n1	9.38	0.6	0.9	0.9	0.1	1.7	-0.9	0.0	0.7	0.0	0	0.5	5
coronene	36	1	2	P21/n	9.57	0.0	0.6	0.6	0.4	-0.7	0.4	0.0	0.2	0.0	0	0.2	1
DATT	48	1	1	P21	9.98	0.3	0.6	0.6	0.6	-0.8	0.4	0.0	-0.2	0.0	12	0.4	8
DBTTF	26	1	2	P21/c	11.69	0.3	0.3	0.4	0.3	-0.4	0.3	0.0	-0.1	0.0	0	0.5	1
dibenzoindigo	42	1	2	P21/n	9.59	0.1	3.2	3.2	-1.2	1.9	-0.5	0.0	0.5	0.0	0	1.5	10
DNBDT	42	1	2	P21/n	9.94	0.4	0.3	0.4	0.4	-0.2	0.3	0.0	-0.0	0.0	0	0.2	4
DNTT	36	1	1	P21	10.39	0.6	0.4	0.5	0.3	-0.2	0.5	0.0	0.0	0.0	5	0.3	5
hexacene	42	2	2	P-1	9.05	-0.3	1.2	1.2	0.2	-1.1	0.3	-1.0	-0.9	-0.1	0	1.2	3
HMTTF	28	1	1	P21/c	10.18	0.2	0.2	0.2	0.0	-0.0	0.3	0.0	-0.1	0.0	12	0.2	7
ICZb	32	1	2	P21/c	9.16	0.4	0.8	0.8	0.1	0.9	-0.7	0.0	-0.8	0.0	0	0.7	4
ICZbCl2c	32	1	2	P21/c	10.34	0.0	0.8	0.8	0.1	1.1	-1.2	0.0	-0.3	0.0	0	1.5	7
IF12b	34	1	2	P21/n	9.17	0.6	1.1	1.0	-0.1	1.3	-0.6	0.0	0.8	0.0	0	0.6	5
IF21a	34	1	1	P21/c	9.58	0.7	0.7	0.8	-0.9	1.2	0.3	0.0	0.5	0.0	7	0.7	3
indigo	30	1	2	P21/n	9.59	0.4	0.8	0.8	0.1	0.8	-0.8	0.0	-0.4	0.0	0	0.5	3
indigoCl2	30	1	2	P21/c	11.01	0.4	0.6	0.5	-0.3	1.1	-0.2	0.0	0.2	0.0	0	0.3	3
naphthalene	18	1	2	P21/c	9.41	1.4	0.2	0.5	0.2	0.7	0.4	0.0	-0.1	0.0	0	0.3	0
NDFPh	44	1	2	P21/c	9.86	0.4	0.4	0.5	0.4	-0.5	0.4	0.0	-0.1	0.0	0	0.3	7
NDI	26	2	2	P-1	10.07	0.1	0.9	0.9	1.3	-0.4	-0.7	-0.1	-0.1	-0.3	0	0.8	7
NDT	24	2	2	P21/c	10.92	0.6	3.0	3.2	3.0	-2.4	-0.1	0.0	-0.6	0.0	0	2.6	4
oligothiophene4	30	1	2	P21/a	11.73	0.7	1.3	1.4	-1.3	0.6	1.2	0.0	-0.6	0.0	0	1.0	4
oligothiophene6	44	1	2	P21/a	11.70	0.1	1.1	1.1	-1.3	0.6	0.8	0.0	-0.1	0.0	0	1.3	13
OPV3CN	42	1	2	Pbca	10.12	0.2	0.4	0.5	0.1	-0.3	0.3	0.0	0.0	0.0	0	0.7	29
PBBTZ	30	1	1	P21/n	10.57	0.2	0.2	0.3	-0.1	0.2	0.1	0.0	0.1	0.0	2	0.1	2
PDI	40	1	2	P21/n	9.64	0.3	0.5	0.5	-0.0	0.5	-0.2	0.0	-0.2	0.0	0	0.2	3
pentacene	36	2	2	P-1	9.17	0.3	0.8	0.8	-0.7	1.1	0.1	-0.5	-0.4	0.2	0	0.7	3
pentaceneN2Cl4	34	1	2	P21/n	11.88	0.8	0.8	1.0	-0.4	1.3	-0.0	0.0	-0.1	0.0	0	0.2	5
pentaceneN4O2	32	1	2	P21/n	9.92	-0.0	0.3	0.3	-0.4	0.4	-0.1	0.0	0.1	0.0	0	0.2	3
perylene	32	1	2	P21/c	9.16	1.1	2.1	2.0	0.2	2.9	-2.0	0.0	-0.6	0.0	0	1.3	3
phenazine	22	1	2	P21/n	9.75	0.9	0.4	0.5	0.7	0.3	-0.1	0.0	0.3	0.0	0	1.6	2
PTCDA	38	1	2	P21/c	10.10	-0.1	0.5	0.5	-0.3	0.4	-0.3	0.0	-0.4	0.0	0	0.7	3
pyrene	26	1	1	P21/a	9.60	0.3	1.4	1.4	-1.5	1.2	0.5	0.0	-0.6	0.0	10	1.2	2
quinacridone	36	1	2	P21/c	9.47	0.9	1.5	1.7	1.6	-0.7	0.3	0.0	0.8	0.0	0	0.9	6
spirobidibenzosilole	41	1	2	P41212	10.43	0.6	0.2	0.4	0.1	0.1	0.3	0.0	0.0	0.0	3	0.3	12
spirobifluorene	41	1	1	P21/c	10.11	0.3	0.2	0.2	-0.0	0.4	-0.1	0.0	0.0	0.0	17	0.2	7
stilbene	26	2	2	P21/a	9.37	0.8	1.1	1.1	1.3	0.2	-0.4	0.0	0.4	0.0	0	0.5	3
TCNQ	20	1	2	C2/c	12.42	0.6	0.6	0.5	0.4	0.6	-0.3	0.0	0.4	0.0	0	0.9	3
TCNQ-F2	20	1	4	C2/m	12.84	0.1	0.4	0.4	0.1	-0.3	0.5	0.0	0.3	0.0	0	0.2	3
TCNQ-F4	20	1	2	Pbca	13.90	-0.5	1.1	1.1	-0.9	-0.8	1.1	0.0	0.0	0.0	0	0.7	6
tetracene	30	2	2	P-1	9.30	0.6	1.1	1.1	1.2	-1.0	0.4	0.7	-0.4	-0.0	0	0.7	1
thiazolothiazolePh	30	1	2	P21/c	10.70	1.0	0.8	1.3	-0.7	0.7	0.8	0.0	-0.4	0.0	0	1.1	5
TPP	34	1	1	P21/c	10.07	0.4	0.4	0.4	-0.0	-0.3	0.6	0.0	-0.1	0.0	16	0.4	20
triazine	9	1	2	C2/c	10.20	-0.8	0.2	0.4	-0.3	-0.2	-0.6	0.0	-0.2	0.0	0	0.1	1
triphenylbenzene	42	1	1	Pna21	9.55	0.7	0.3	0.5	-0.2	0.5	0.4	0.0	0.0	0.0	5	0.1	8
triphenylene	30	1	1	P212121	9.25	0.0	0.4	0.4	-0.2	-0.2	0.4	0.0	0.0	0.0	11	0.4	3
triphenyltriazine	39	1	1	P21/c	9.43	0.4	0.4	0.6	0.4	-0.3	0.3	0.0	-0.0	0.0	9	0.1	7
triptycene	34	1	1	P212121	9.77	1.0	0.1	0.3	0.3	0.5	0.2	0.0	0.0	0.0	6	0.1	3
TTAa	36	1	2	P21/n	11.27	0.5	0.2	0.3	-0.1	0.4	0.2	0.0	0.2	0.0	0	0.2	2
TTAb	36	1	2	P21/n	11.25	0.5	0.5	0.4	0.0	0.7	-0.3	0.0	-0.3	0.0	0	0.2	2
TTF	14	1	2	P21/c	13.51	0.4	0.9	1.0	0.1	-0.5	0.5	0.0	-0.9	0.0	0	1.2	3
TTPa	34	1	2	P21/n	11.67	0.0	0.3	0.3	-0.1	0.2	-0.1	0.0	0.2	0.0	0	0.2	3
TTPb	34	2	2	P21/a	11.83	-0.0	0.4	0.3	0.3	-0.4	0.1	0.0	0.1	0.0	0	0.4	3
TTPc	34	1	1	P21	11.92	0.5	0.5	0.7	0.2	-0.5	0.8	0.0	-0.1	0.0	29	0.4	13
TTTT	18	1	2	P21/n	12.70	0.3	0.6	0.7	0.6	-0.4	0.3	0.0	0.4	0.0	0	0.2	1

Table S16: Smaller cutoff with unconstrained relaxation for PBE-D3 [URL].

Unconstrained relaxation of unit cell (cg3) at 600eV compared to 900eV and volume scan at 600eV:

system	600cg3	900cg3	600vs	
anthraquinone	9.443	9.669	9.733	
benzene	9.270	9.544	9.575	
benzeneF6	12.575	13.261	13.395	* difference is 6%
naphthalene	9.163	9.413	9.449	
pentacene	8.968	9.169	9.242	
TCNQ	12.184	12.424	12.507	
triazine	9.966	10.202	10.227	
triphenylbenzene	9.314	9.549	9.597	

Table S17: Gradients at optimized geometry: PBE-D3paw900 [URL].

cg3, N=67, gradient in meV/Ao, 2023-04-10

	optimization			SP		finerSP	
	nopt	|G|	maxG	|G|	maxG	|G|	maxG
anthracene	1	1	1	0	1	0	1
anthraquinone	75	1	1	1	1	1	1
benzene	5	1	1	1	1	1	1
benzeneF6	9	1	1	1	1	1	1
benzeneO2	7	1	1	1	1	1	1
benzodithiopheneO2	96	1	1	1	1	1	1
benzotrithiophene	93	0	1	1	1	1	1
benzotrithiopheneA	1	0	1	1	1	1	1
bithiophene	1	1	1	1	1	1	1
BODIPY	8	0	1	1	1	1	1
BTBT	41	1	1	1	1	1	1
C60	5	1	1	1	1	1	1
carbazole	1	0	1	0	1	0	1
carbazoleNO2b	1	0	1	0	1	0	1
chrysene	35	1	1	1	1	1	1
coronene	1	1	1	1	1	1	1
DATT	1	1	1	1	1	1	1
DBTTF	5	1	1	1	1	1	1
dibenzoindigo	9	0	1	0	1	0	1
DNBDT	1	0	1	0	1	0	1
DNTT	4	1	1	1	1	1	1
hexacene	1	0	1	1	1	1	1
HMTTF	39	1	1	1	1	1	1
ICZb	3	1	1	1	1	1	1
ICZbCl2c	74	0	1	1	1	1	1
IF12b	1	1	1	1	1	1	1
IF21a	1	1	1	1	1	1	1
indigo	39	1	1	1	1	1	1
indigoCl2	1	1	1	1	1	1	1
naphthalene	1	1	1	1	1	1	1
NDFPh	1	1	1	1	1	1	1
NDI	5	0	1	0	1	0	1
NDT	1	1	1	1	1	1	1
oligothiophene4	6	1	1	1	1	1	1
oligothiophene6	27	1	1	1	1	1	1
OPV3CN	5	1	1	1	1	1	1
PBBTZ	81	0	1	0	1	1	1
PDI	1	1	1	1	1	1	1
pentacene	6	0	1	0	1	0	1
pentaceneN2Cl4	3	1	1	1	1	1	1
pentaceneN4O2	1	1	1	1	1	1	1
perylene	1	1	1	1	1	1	1
phenazine	47	1	1	1	1	1	1
PTCDA	36	0	1	0	1	0	1
pyrene	8	1	1	1	1	1	1
quinacridone	54	1	1	1	1	1	1
spirobidibenzosilole	47	0	1	1	1	1	1
spirobifluorene	7	1	1	1	1	1	1
stilbene	1	1	1	1	1	1	1
TCNQ	1	0	1	1	1	1	1
TCNQ-F2	6	1	1	1	1	1	1
TCNQ-F4	8	0	1	1	1	1	1
tetracene	83	0	1	1	1	1	1
thiazolothiazolePh	9	0	1	0	1	0	1
TPP	4	1	1	1	1	1	1
triazine	34	1	1	1	1	1	1
triphenylbenzene	12	1	1	1	1	1	1
triphenylene	3	0	1	0	1	0	1
triphenyltriazine	1	1	1	1	1	1	1
triptycene	1	0	1	0	1	0	1
TTAa	7	1	1	1	1	1	1
TTAb	1	1	1	1	1	1	1
TTF	69	1	1	1	1	1	1
TTPa	3	0	1	1	1	1	1
TTPb	7	1	1	1	1	1	1
TTPc	58	1	1	1	1	1	1
TTTT	9	1	1	1	1	1	1

Table S18: Comparison of different k-grids (PBE-D3paw900) [URL].

```
Comparison of different k-grids
method=PBE-D3paw900
exp=relaxed from experimental geometry
100812=10x8x12 k-grid

              V       a       b       c   alpha    beta   gamma
--- anthraquinone (P21/c) -------------------------------------
 482       9.669   7.834   3.862  15.713   90     102.53   90
 241       9.675   7.837   3.863  15.715   90     102.55   90
 222       9.902   7.677   4.167  15.392   90     105.14   90
 221       9.906   7.677   4.168  15.394   90     105.13   90
 111       9.270   4.776   6.102  17.518   90     119.36   90

--- benzene (Pbca) --------------------------------------------
 100812    9.544   7.322   9.328   6.708   90      90      90
 646       9.544   7.322   9.328   6.708   90      90      90
 323       9.544   7.322   9.328   6.708   90      90      90
 222       9.543   7.321   9.326   6.708   90      90      90
 222(exp)  9.551   7.320   9.326   6.715   90      90      90
 111       9.944  10.976   8.995   4.835   90      90      90

--- benzeneF6 (P21/n) -----------------------------------------
 642      13.261   5.976   9.368  17.096   90      94.01   90
 222      13.269   5.980   9.405  17.030   90      94.11   90
 221      13.272   5.976   9.375  17.100   90      94.06   90
 111      12.046   4.274   9.831  21.744   90     108.30   90

--- BTBT (P21/c) ----------------------------------------------
 365      10.915  11.790   5.887   7.844   90     105.78   90
 222      10.900  11.788   5.866   7.862   90     105.76   90
 111      10.030  12.006   4.583   9.179   90     107.59   90

--- naphthalene (P21/c) ---------------------------------------
 454       9.413   7.812   5.902   8.064   90     114.29   90
 222       9.417   7.801   5.919   8.048   90     114.17   90
 111       8.904   9.941   4.583   8.683   90     125.88   90

--- NDI (P-1) -------------------------------------------------
 444      10.068   7.527   8.225   9.066   89.77   71.61   79.89
 222      10.082   7.526   8.233   9.067   89.79   71.71   79.84
 111       9.911   8.022   8.388   8.211   89.90   74.20   76.34

--- pentacene (P-1) -------------------------------------------
 542       9.169   6.252   7.620  14.291   77.08   88.79   84.17
 222       9.167   6.265   7.613  14.271   77.09   88.90   84.12
 221       9.184   6.257   7.625  14.308   76.89   88.93   84.11
 111       8.173   4.360   8.796  16.838   71.29   75.03   89.99

--- TCNQ (C2/c:P) ---------------------------------------------
 662      12.424   8.848   6.876  16.483   90      97.59   90
 222      12.440   8.886   7.008  16.120   90      97.54   90
 111       8.418   7.682   4.723  18.807   90      99.28   90

--- TCNQ-F2 (C2/m:P) ------------------------------------------
 774      12.837  10.199   5.951   8.817   90     106.37   90
 222      12.884  10.201   5.954   8.826   90     105.99   90
 111      10.222   8.353   5.534   8.965   90      99.40   90
```

S6 Additional figures and tables for approximate DFT methods

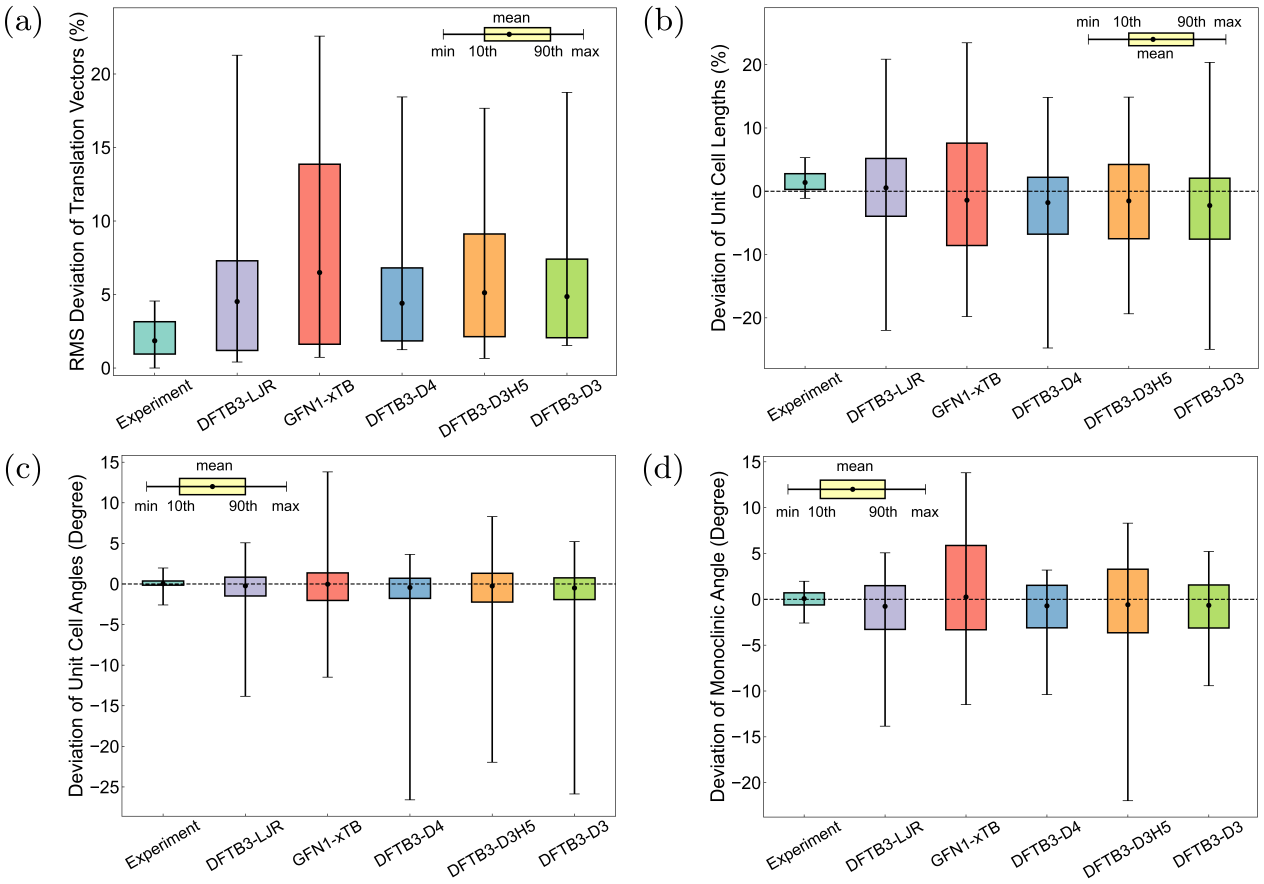

Figure S10: Benchmark of approximate DFT methods against DFT (r^2SCAN-D3) reference. (a) The RMS deviation of translation vectors, (b) deviation of unit cell lengths a, b and c, (c) deviation of unit cell angles α, β and γ, (d) deviation of monoclinic angle β.

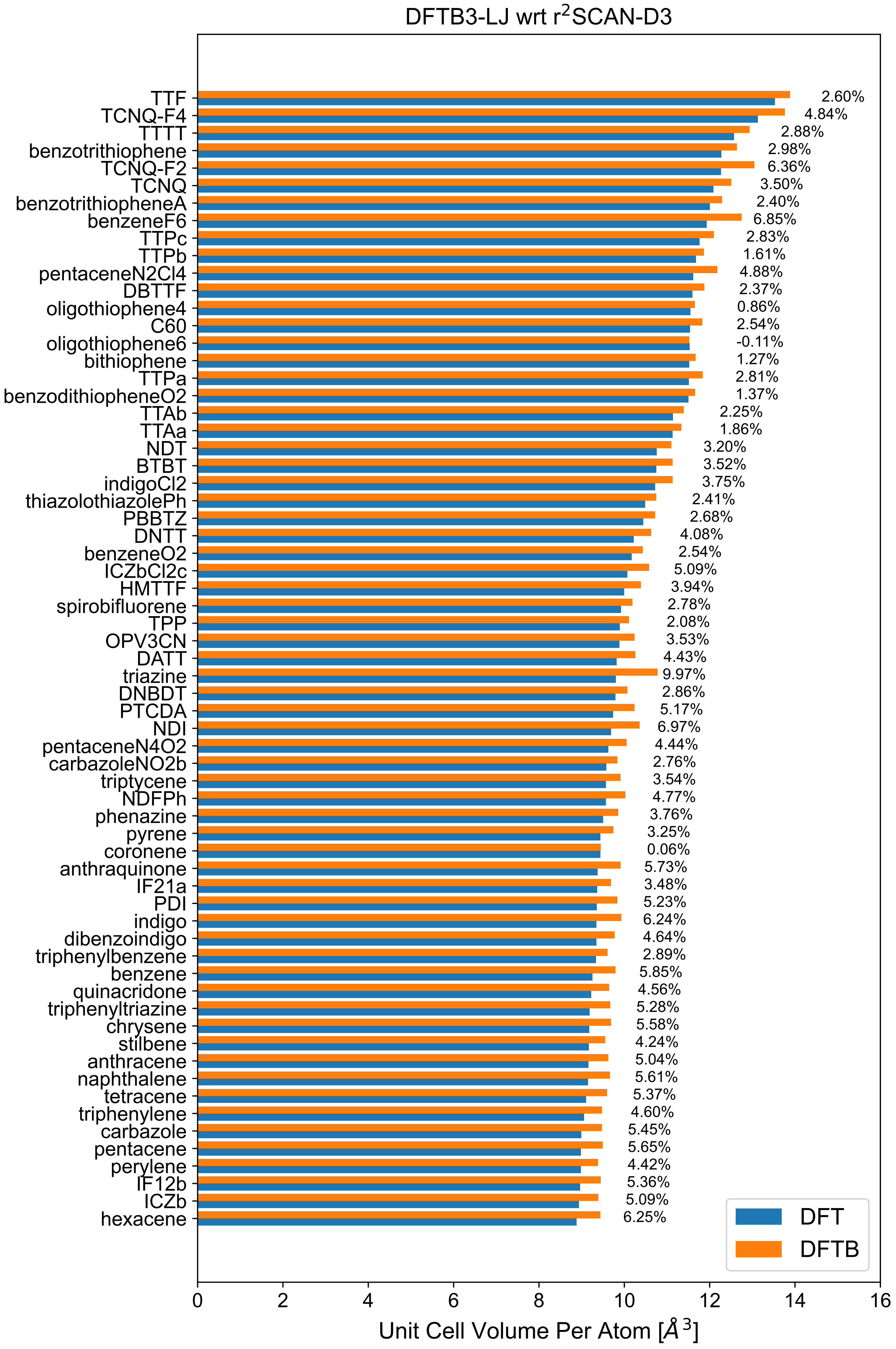

Figure S11: Comparison between DFTB3-LJ with original parameters and DFT reference on unit cell volume. The average error is 3.92%.

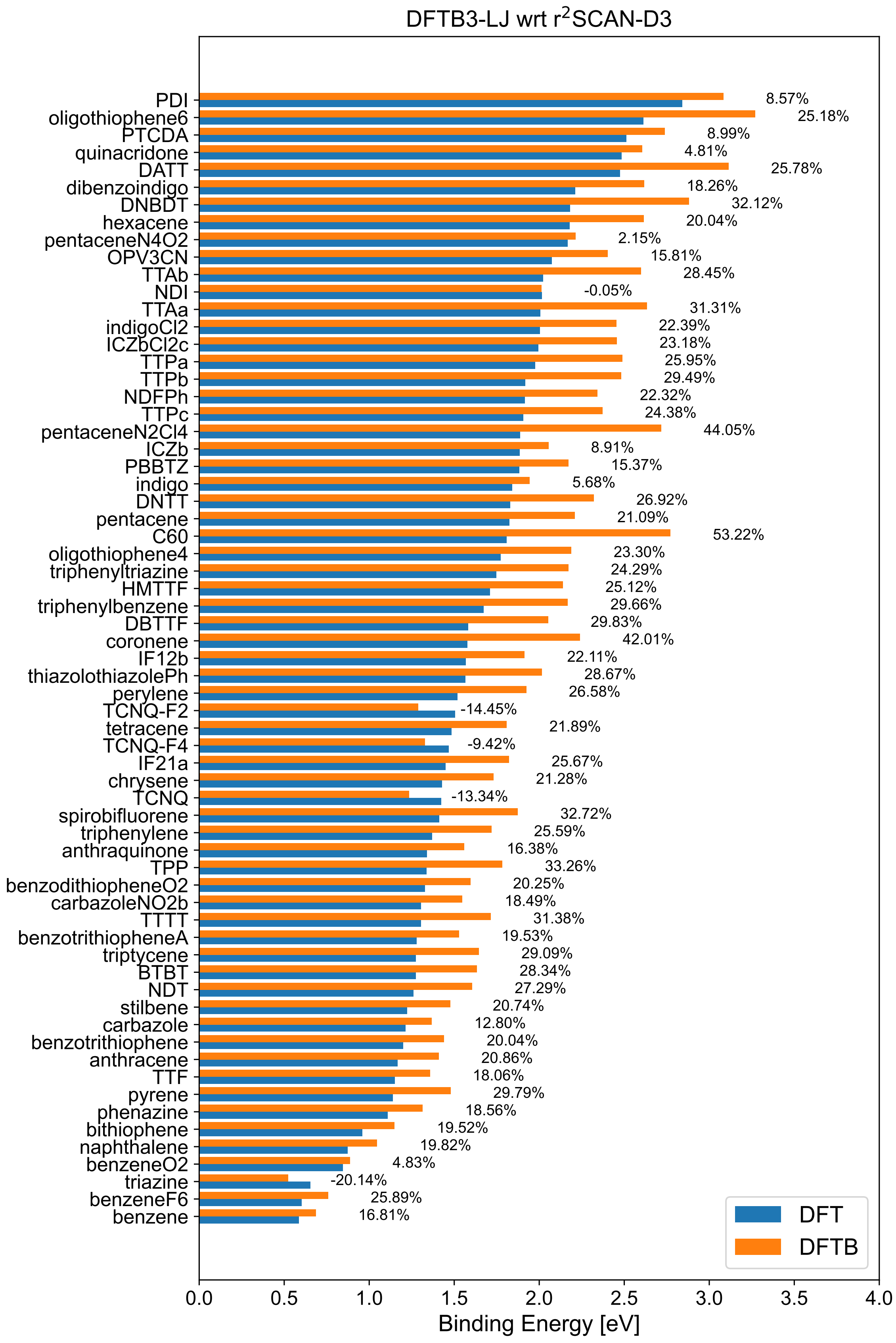

Figure S12: Comparison between DFTB3-LJ with original parameters and DFT reference on binding energy. The average error is 20.30%.

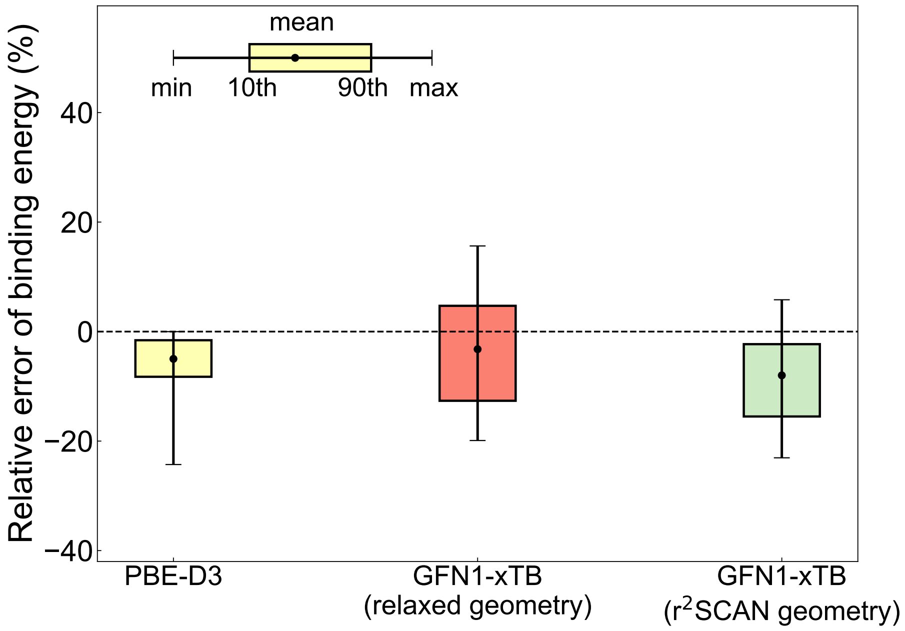

Figure S13: The comparison of binding energies calculated by GFN1-xTB using relaxed geometries and r^{2}SCAN geometries.

Table S19: The groupings of crystals based on chemical features.

Group name and description	Chemical feature	System
C – hydrocarbons	C	anthracene
	C	benzene
	C	C60
	C	chrysene
	C	coronene
	C	hexacene
	C	IF12b
	C	IF21a
	C	naphthalene
	C	pentacene
	C	perylene
	C	pyrene
	C	stilbene
	C	tetracene
	C	triphenylbenzene
	C	triphenylene
	CCsp3	spirobifluorene
	CCsp3	triptycene
CS – molecules with sulfur in aromatic rings	CS	benzotrithiophene
	CS	benzotrithiopheneA
	CS	bithiophene
	CS	BTBT
	CS	DATT
	CS	DBTTF
	CS	DNBDT
	CS	DNTT
	CS	HMTTF
	CS	NDT
	CS	oligothiophene4
	CS	oligothiophene6
	CS	TTAa
	CS	TTAb
	CS	TTF
	CS	TTTT
CN – molecules with nitrogen in aromatic rings	CN	carbazole
	CN	ICZb
	CN	phenazine
	CN	triazine
	CN	triphenyltriazine
CO – molecules with π-conjugated oxygen atoms	C(O)	anthraquinone
	CO	NDFPh
	CO(O)	PTCDA
	C(O)	benzeneO2
CNH(O) – this group contains a wide range of structures incorporating carbon, nitrogen, and hydrogen in their backbone structure, and in some cases carbonyl or nitro substituents	CN(O)	pentaceneN4O2
	CNH(O)	dibenzoindigo
	CNH(O)	indigo
	CNH(O)	NDI
	CNH(O)	PDI
	CNH(O)	quinacridone
	CNH(NO2)	carbazoleNO2b
CNS – structures with carbon, nitrogen, and sulphur	CNS	thiazolothiazolePh
	CNS	TTPa
	CNS	TTPb
	CNS	TTPc
CNBF – structures with C, N, B, and F	CNBF	BODIPY
C(CN) – structures with cyano groups	C(CN)	OPV3CN
	C(CN)	TCNQ
F – molecules with fluorine atoms	C(F)	benzeneF6
	C(CN)F	TCNQ-F2
	C(CN)F	TCNQ-F4
Cl – molecules with chlorine atoms	CNH(O)Cl	indigoCl2
	CNCl	ICZbCl2c
	CNCl	pentaceneN2Cl4
Other molecules	CS(O)	benzodithiopheneO2
	CSNNH	PBBTZ
	CSi	spirobidibenzosilole
	CP	TPP

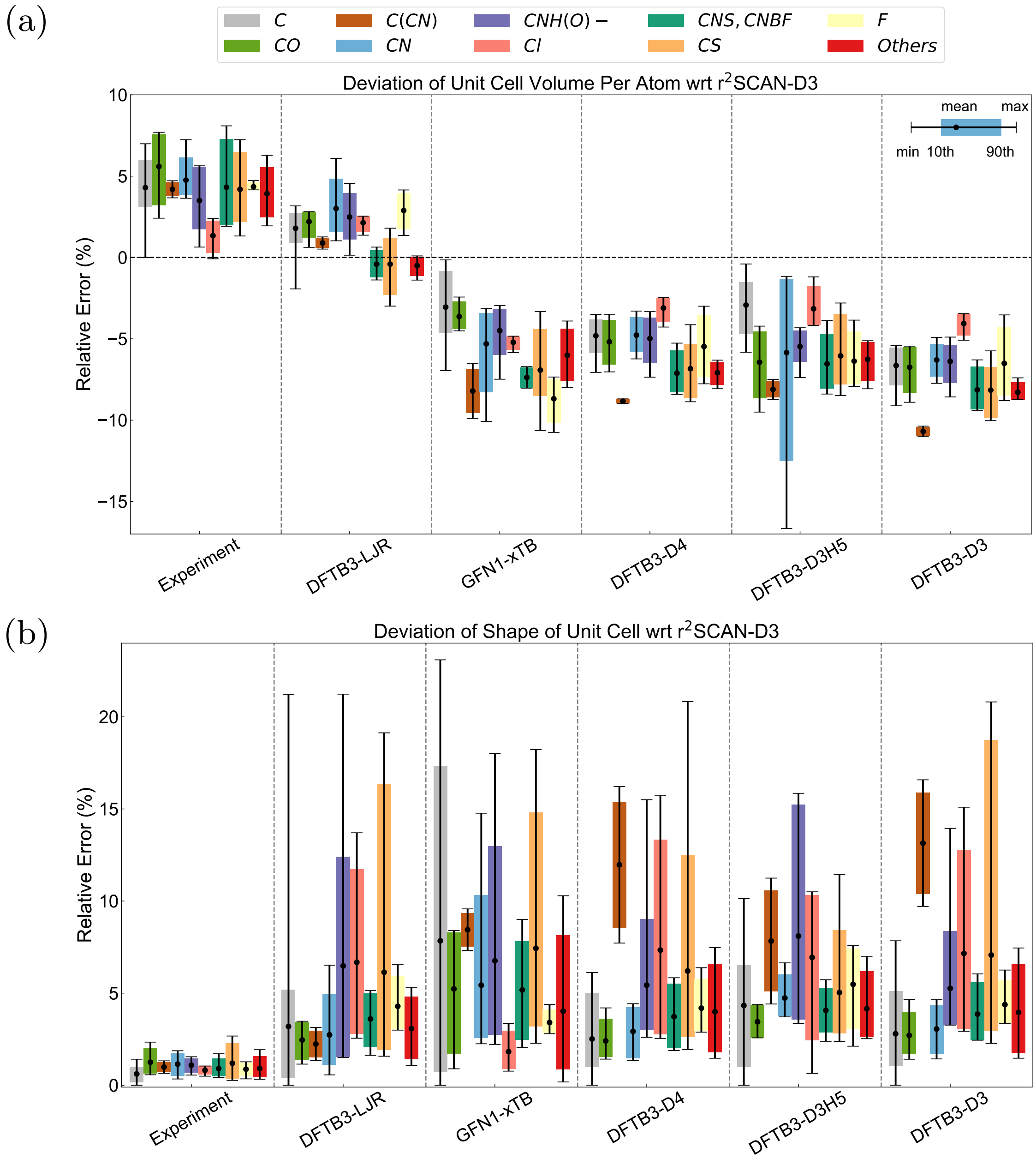

Figure S14: Performance of the approximate DFT methods on different groups of crystals. The deviations of (a) unit cell volume per atom and (b) shape of unit cell.

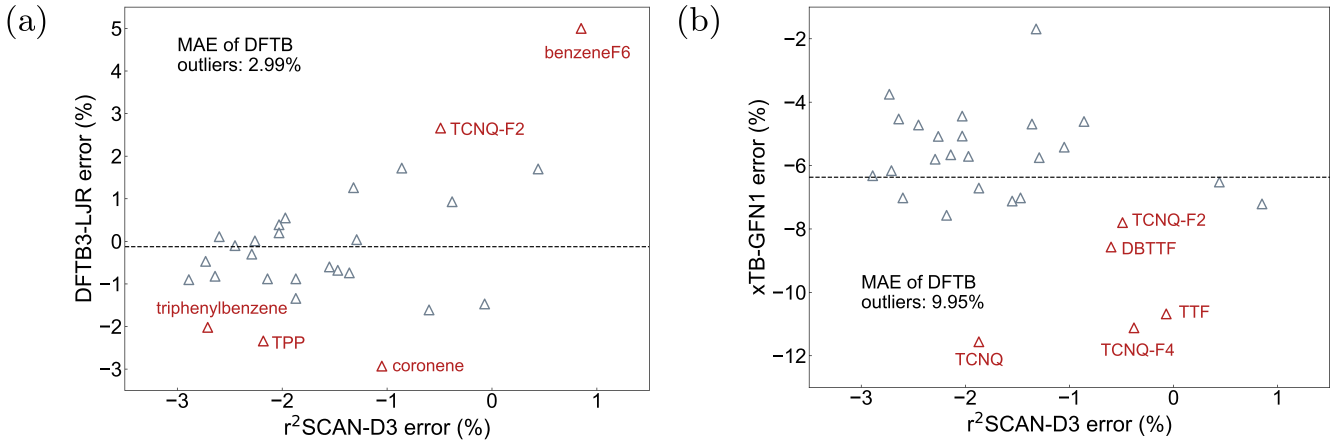

Figure S15: (a) The comparison between the error of r^{2}SCAN-D3 and DFTB3-LJR in terms of unit cell volume per atom, using experimental results with extrapolated volumes (zero temperature) as the reference. (b) The comparison between the error of r^{2}SCAN-D3 and xTB-GFN1 in terms of unit cell volume per atom, using experimental results with extrapolated volumes (zero temperature) as the reference. In the plots, the red triangles represent 5 systems exhibiting the highest absolute errors, which are identified as the outliers for the two approximate DFT methods. The gray triangles indicate the remaining systems. The black dashed line displays the mean error of the DFTB3-LJR/xTB-GFN1 method. For comparison, the mean absolute error (MAE) of the 5 outliers for the DFTB3-LJR and the xTB-GFN1 method is 2.99% and 9.95%, respectively.
